# Supplementary material for: Evaluation of a clinical decision rule to guide antibiotic prescription in children with suspected lower respiratory tract infection in The Netherlands: A stepped-wedge cluster randomised trial
Source: PLoS Med. 2020 Jan 31;17(1):e1003034. doi: 10.1371/journal.pmed.1003034 (PMC6993966; doi:10.1371/journal.pmed.1003034)
Supplement: S2 Table — (PDF) [file pmed.1003034.s002.pdf]

Comparison of included and non-included children

	Included<br>n=999	Non-included<br>n=677
**General characteristics**		
Male sex	610/999 (61%)	432/674 (64%)
Age in months*	17 (9 - 30)	12 (6 - 24)
Triage level*		
- *Immediate or very urgent*	488/908 (54%)	251/641 (39%)
- *Urgent*	267/908 (29%)	266/641 (41%)
- *Standard or non-urgent*	83/908 (9%)	124/641 (19%)
Antibiotic prescription*	280/999 (28%)	106/677 (16%)
Hospitalization*	510/999 (51%)	242/677 (36%)

* significant difference between included and non-included children, based on t-test for continuous outcomes and based on chi-squared test for categorical outcomes.
