# Supplementary material for: Evaluation of a clinical decision rule to guide antibiotic prescription in children with suspected lower respiratory tract infection in The Netherlands: A stepped-wedge cluster randomised trial
Source: PLoS Med. 2020 Jan 31;17(1):e1003034. doi: 10.1371/journal.pmed.1003034 (PMC6993966; doi:10.1371/journal.pmed.1003034)
Supplement: S3 Table — (PDF) [file pmed.1003034.s003.pdf]

## Baseline characteristics per hospital

| | Overall n = 999 | | Hospital A n = 196 | | Hospital B n = 85 | | Hospital C n = 46 | | Hospital D n = 164 | | Hospital E n = 31 | | Hospital F n = 98 | | Hospital G n =211 | | Hospital H n = 168 | |
|---|---|---|---|---|---|---|---|---|---|---|---|---|---|---|---|---|---|---|
| | n / median | %/IQR | *General hospital* | | *General hospital* | | *Tertiary hospital* | | *General hospital* | | *General hospital* | | *General hospital* | | *General hospital* | | *General hospital* | |
| **General characteristics** | | | | | | | | | | | | | | | | | | |
| Male sex | 611 | 61% | 114 | 58% | 58 | 68% | 31 | 67% | 97 | 59% | 17 | 55% | 54 | 55% | 138 | 65% | 101 | 60% |
| Age in months | 17 | 9 - 30 | 13 | 8 - 25 | 18 | 10 - 33 | 22 | 11 - 37 | 15 | 8 - 30 | 19 | 12 - 27 | 14 | 9 - 26 | 20 | 10 - 34 | 16 | 9 - 28 |
| Season | | | | | | | | | | | | | | | | | | |
| - *Spring* | 190 | 19% | 39 | 20% | 16 | 19% | 7 | 15% | 29 | 18% | 3 | 10% | 16 | 16% | 38 | 18% | 42 | 25% |
| - *Summer* | 104 | 10% | 21 | 11% | 4 | 5% | 4 | 9% | 16 | 10% | 2 | 6% | 9 | 9% | 19 | 9% | 29 | 17% |
| - *Autumn* | 287 | 29% | 43 | 22% | 22 | 26% | 18 | 39% | 56 | 34% | 12 | 39% | 32 | 33% | 70 | 33% | 34 | 20% |
| - *Winter* | 431 | 43% | 93 | 47% | 43 | 51% | 17 | 37% | 66 | 40% | 14 | 45% | 41 | 42% | 84 | 40% | 63 | 38% |
| Way of referral | | | | | | | | | | | | | | | | | | |
| - *General practitioner* | 739 | 74% | 148 | 76% | 56 | 66% | 19 | 41% | 137 | 84% | 18 | 58% | 84 | 86% | 136 | 64% | 138 | 82% |
| - *Self* | 111 | 11% | 11 | 6% | 4 | 5% | 11 | 24% | 8 | 5% | 1 | 3% | 3 | 3% | 61 | 29% | 12 | 7% |
| - *Other* | 110 | 11% | 30 | 15% | 7 | 8% | 11 | 24% | 17 | 10% | 12 | 39% | 11 | 11% | 13 | 6% | 9 | 5% |
| **Signs and symptoms** | | | | | | | | | | | | | | | | | | |
| Ill appearance | 359 | 36% | 80 | 41% | 25 | 29% | 12 | 26% | 89 | 54% | 7 | 23% | 37 | 38% | 68 | 32% | 40 | 24% |
| Duration of fever in days | 2 | 1-4 | 2 | 1 - 4 | 3 | 1 - 4 | 3 | 1 - 5 | 2 | 1 - 3 | 2 | 1 - 4 | 2 | 1 - 4 | 2 | 1 - 3 | 2 | 1 - 3 |
| Temperature | 38.8 | 38.1 - 39.5 | 38.9 | 38.2 - 39.5 | 38.8 | 38.1 - 39.1 | 39.1 | 38 - 39.7 | 38.9 | 38.1 - 39.6 | 38.5 | 37.5 - 39.2 | 38.6 | 37.8 - 39.2 | 39.0 | 38.5 - 39.6 | 38.6 | 38 - 39.4 |
| Hypoxia (sat <94%) | 218 | 22% | 36 | 18% | 14 | 16% | 10 | 22% | 44 | 27% | 3 | 10% | 30 | 31% | 56 | 27% | 25 | 15% |
| Tachycardia | 691 | 69% | 124 | 63% | 60 | 71% | 32 | 70% | 123 | 75% | 23 | 74% | 71 | 72% | 133 | 63% | 124 | 74% |
| Tachypnea | 804 | 80% | 140 | 71% | 69 | 81% | 35 | 76% | 139 | 85% | 18 | 58% | 87 | 89% | 169 | 80% | 145 | 86% |
| Retractions present | 614 | 61% | 106 | 54% | 57 | 67% | 20 | 43% | 114 | 70% | 17 | 55% | 59 | 60% | 150 | 71% | 90 | 54% |
| Dyspnea present | 723 | 72% | 133 | 68% | 64 | 75% | 27 | 59% | 138 | 84% | 21 | 68% | 59 | 60% | 161 | 76% | 119 | 71% |
| Wheezing | 366 | 37% | 60 | 31% | 31 | 36% | 11 | 24% | 69 | 42% | 11 | 35% | 21 | 21% | 92 | 44% | 70 | 42% |
| Prolonged capillary refill (≥2 seconds) | 116 | 12% | 21 | 11% | 4 | 5% | 4 | 9% | 33 | 20% | 0 | 0% | 15 | 15% | 31 | 15% | 7 | 4% |
| **Diagnostics and treatment** | | | | | | | | | | | | | | | | | | |
| CRP performed | 756 | 76% | 136 | 69% | 78 | 92% | 39 | 85% | 90 | 55% | 27 | 87% | 84 | 86% | 137 | 65% | 165 | 98% |
| CRP in mg/L | 18 | 7 - 41 | 14 | 7 - 28 | 20 | 10 - 47 | 17 | 5 - 33 | 21 | 7 - 50 | 9 | 5 - 33 | 26 | 8 - 59 | 22 | 8 - 38 | 18 | 5 - 42 |
| Chest x-ray performed | 159 | 16% | 17 | 9% | 10 | 12% | 6 | 13% | 37 | 23% | 14 | 45% | 21 | 21% | 37 | 18% | 16 | 10% |
| Oxygen therapy | 415 | 41% | 65 | 33% | 30 | 35% | 14 | 30% | 102 | 62% | 9 | 29% | 47 | 48% | 80 | 38% | 68 | 40% |
| Antibiotic prescription | 281 | 28% | 36 | 18% | 17 | 20% | 17 | 37% | 65 | 40% | 6 | 19% | 37 | 38% | 63 | 30% | 39 | 23% |
| Hospitalization | 511 | 51% | 88 | 45% | 39 | 46% | 19 | 41% | 119 | 73% | 10 | 32% | 58 | 59% | 91 | 43% | 86 | 51% |
