# Supplementary material for: Evaluation of a clinical decision rule to guide antibiotic prescription in children with suspected lower respiratory tract infection in The Netherlands: A stepped-wedge cluster randomised trial
Source: PLoS Med. 2020 Jan 31;17(1):e1003034. doi: 10.1371/journal.pmed.1003034 (PMC6993966; doi:10.1371/journal.pmed.1003034)
Supplement: S4 Table — (PDF) [file pmed.1003034.s004.pdf]

Antibiotic prescription per hospital and per time period

Time period	1	2	3	4	5	6	7	8	9
**Hospital**	n/N (%)	n/N (%)	n/N (%)	n/N (%)	n/N (%)	n/N (%)	n/N (%)	n/N (%)	n/N (%)
**A**	11/69 (16%)	0/7 (0%)	2/9 (22%)	2/12 (17%)	5/28 (18%)	5/15 (33%)	4/20 (5%)	5/17 (29%)	2/19 (11%)
**B**	8/33 (24%)	0/2 (0%)	1/3 (33%)	0/6 (0%)	1/16 (6%)	2/15 (13%)	1/3 (33%)	1/1 (100%)	3/6 (50%)
**C**	10/27 (37%)	0/1 (0%)	1/1 (100%)	1/5 (20%)	0/3 (0%)	0/1 (0%)	2/3 (67%)	1/2 (50%)	2/3 (67%)
**D**	35/74 (47%)	3/6 (50%)	1/8 (13%)	3/7 (43%)	6/23 (26%)	3/9 (33%)	0/4 (0%)	3/5 (60%)	11/28 (39%)
**E**	0/0 (0%)	0/0 (0%)	1/5 (20%)	0/3 (0%)	3/12 (25%)	0/2 (0%)	0/3 (0%)	0/1 (0%)	2/5 (40%)
**F**	23/52 (44%)	0/0 (0%)	1/4 (25%)	4/8 (50%)	0/9 (0%)	2/9 (22%)	2/5 (40%)	0/0 (0%)	5/11 (45%)
**G**	1/1 (100%)	3/17 (18%)	1/12 (8%)	5/23 (22%)	19/51 (37%)	6/20 (30%)	5/20 (25%)	5/10 (50%)	18/57 (32%)
**H**	27/71 (38%)	0/3 (0%)	0/2 (0%)	1/6 (17%)	1/10 (10%)	2/11 (18%)	1/13 (8%)	1/7 (14%)	6/45 (13%)

Footnote: dark blue = pre-intervention period; light blue = intervention period.
