# Supplementary material for: Evaluation of a clinical decision rule to guide antibiotic prescription in children with suspected lower respiratory tract infection in The Netherlands: A stepped-wedge cluster randomised trial
Source: PLoS Med. 2020 Jan 31;17(1):e1003034. doi: 10.1371/journal.pmed.1003034 (PMC6993966; doi:10.1371/journal.pmed.1003034)
Supplement: S5 Table — (PDF) [file pmed.1003034.s005.pdf]

Detailed outcomes per risk group

	Pre-intervention n / N (%)	Intervention n / N (%)
**Low-intermediate risk population (≤10%)**		
Antibiotic prescription*	29/172 (17%)	15/234 (6%)
Strategy failure*	29/159 (18%)	39/218 (18%)
Strategy failure: reasons		
- *Secondary antibiotic prescription*	7/159 (4%)	20/218 (9%)
- *Changed antibiotics during follow-up*	1/159 (1%)	0/218 (0%)
- *Secondary hospitalization*	7/159 (4%)	11/218 (5%)
- *Oxygen need at day 7*	3/159 (2%)	0/218 (0%)
- *Fever at day 7*	11/159 (7%)	8/218 (4%)
**High-risk population (>10%)**		
Antibiotic prescription*	75/159 (47%)	83/140 (59%)
Strategy failure*	42/155 (27%)	20/136 (15%)
Strategy failure: reasons		
- *Secondary antibiotic prescription*	18/155 (12%)	8/136 (6%)
- *Changed antibiotics during follow-up*	6/155 (4%)	5/136 (4%)
- *Secondary hospitalization*	3/155 (2%)	2/136 (1%)
- *Oxygen need at day 7*	2/155 (1%)	1/136 (1%)
- *Fever at day 7*	13/155 (8%)	4/136 (3%)
**Feverkidstool missing†**		
Antibiotic prescription	75/266 (28%)	3/28 (11%)
Strategy failure	60/258 (23%)	2/27 (7%)
Strategy failure: reasons		
- *Secondary antibiotic prescription*	20/258 (8%)	1/27 (4%)
- *Changed antibiotics during follow-up*	7/258 (3%)	0/27 (0%)
- *Secondary hospitalization*	6/258 (2%)	0/27 (0%)
- *Oxygen need at day 7*	4/258 (2%)	0/27 (0%)
- *Fever at day 7*	23/258 (9%)	1/27 (4%)

Footnote:
* The pre-intervention and intervention populations in a stepped-wedge trial cannot be directly compared, but should be adjusted for a secular time-trend;(1) the adjusted analyses of the primary outcomes antibiotic prescription and strategy failure are presented in table 2 of the manuscript.
† In these children no risk could be calculated due to missing Feverkidstool variables, therefore these populations cannot be compared.

1. Hemming K, Haines TP, Chilton PJ, Girling AJ, Lilford RJ. The stepped wedge cluster randomised trial: rationale, design, analysis, and reporting. Bmj. 2015;350:h391.
