# Supplementary material for: Evaluation of a clinical decision rule to guide antibiotic prescription in children with suspected lower respiratory tract infection in The Netherlands: A stepped-wedge cluster randomised trial
Source: PLoS Med. 2020 Jan 31;17(1):e1003034. doi: 10.1371/journal.pmed.1003034 (PMC6993966; doi:10.1371/journal.pmed.1003034)
Supplement: S1 Text — (PDF) [file pmed.1003034.s006.pdf]

STRAP – Study to Reduce Antibiotic prescription in childhood Pneumonia

Study to Reduce Antibiotic prescription in childhood Pneumonia: implementation of a validated decision rule

PROTOCOL TITLE ‘Study to Reduce Antibiotic prescription in childhood Pneumonia: implementation of a validated decision rule’

**Protocol ID**	**Study to Reduce Antibiotic prescription in childhood Pneumonia: implementation of a validated decision rule**
**Short title**	**STRAP – Study to Reduce Antibiotic prescription in childhood Pneumonia**
**EudraCT number**	***NA***
**Version**	**7**
**Date**	**20 August 2018**
**Coordinating investigator/project leader**	***R. Oostenbrink, MD, PhD***<br>***R.oostenbrink@erasmusmc.nl***<br>***Erasmus MC – Sophia, dept general pediatrics, room Sp 1549, Wytemaweg 80, 3015 CN Rotterdam, The Netherlands***<br>***+31107036742***
**Principal investigator(s) per site**	***Erasmus MC: R. Oostenbrink, MD, PhD***<br>***Flevo ziekenhuis, Almere: A. van Wermeskerken, MD***<br>***Maasstad Ziekenhuis, Rotterdam: F. Smit, MD***<br>***Sint Franciscus Gasthuis, Rotterdam: G. Tramper-Strangers, MD, PhD***<br>***St. Elisabeth Ziekenhuis, Tilburg: C.C. Obihara, MD, PhD***<br>***Reinier de Graaf Gasthuis, Delft: J. G. Noordzij, MD, PhD***<br>***LangeLand Ziekenhuis, Zoetermeer: J. Punt, MD***<br>***HAGA – Juliana Kinderziekenhuis, Den Haag: G.J.A. Driessen, MD, PhD***
**Sponsor (in Dutch: verrichter/opdrachtgever)**	***NA***
**Subsidising party**	***NA***

**Independent expert (s)**	***I.M. De Kleer, MD, PhD***<br>***Pediatrician/, Erasmus MC – Sophia, dept pediatric infectiology/immunology***<br>***Sp 3570***<br>***Erasmus MC – Sophia,***<br>***Wytemaweg 80, 3015 CN Rotterdam***<br>***Email: i.dekleer@erasmusmc.nl***<br>***Tel: +31107036104***
**Laboratory sites**	***Dr. J.J.A. van Kampen,***<br>***Erasmus MC***<br>***Department of Viroscience***<br>***Wytemaweg 80, 30 15 CN Rotterdam***
**Pharmacy**	***NA***

PROTOCOL SIGNATURE SHEET

Name	Signature	Date
**Sponsor or legal representative:**<br>**Head of Department:**<br>***Prof. Dr. E.H.H.M Rings***<br>***Head of dept. pediatrics***<br>***Erasmus MC - Sophia***		
Dr. R. Oostenbrink, project leader and principle investigator<br>***Paediatrician, Erasmus MC - Sophia***		

TABLE OF CONTENTS

Select whole content (click left mouse button) and push F9

1. INTRODUCTION AND RATIONALE 9
2. OBJECTIVES 10
3. STUDY DESIGN 10
4. STUDY POPULATION 11
4.1 Population (base) 11
4.2 Inclusion criteria 11
4.3 Exclusion criteria 11
4.4 Sample size calculation 11
5. TREATMENT OF SUBJECTS 15
5.1 Investigational product/treatment 15
5.2 Use of co-intervention (if applicable) 19
NA 19
5.3 Escape medication (if applicable) 19
NA 19
6. INVESTIGATIONAL PRODUCT 19
6.1 Investigational product/treatment 19
6.2 Name and description of investigational product(s) 19
6.3 Investigational product/treatment 19
6.4 Summary of findings from non-clinical studies 19
6.5 Summary of findings from clinical studies 19
6.6 Summary of known and potential risks and benefits 19
6.7 Description and justification of route of administration and dosage 19
6.8 Dosages, dosage modifications and method of administration 19
6.9 Preparation and labelling of Investigational Medicinal Product 19
6.10 Drug accountability 19
7. NON-INVESTIGATIONAL PRODUCT 19
NA 19
7.1 Name and description of non-investigational product(s) 19
7.2 Summary of findings from non-clinical studies 19
7.3 Summary of findings from clinical studies 19
7.4 Summary of known and potential risks and benefits 19
7.5 Description and justification of route of administration and dosage 19
7.6 Dosages, dosage modifications and method of administration 19
7.7 Preparation and labelling of Non Investigational Medicinal Product 19
7.8 Drug accountability 19
8. METHODS 20
8.1 Study parameters/endpoints 20
8.1.1 Main study parameter/endpoint 20
8.1.2 Other study parameters 20
8.2 Randomisation, blinding and treatment allocation 20
8.3 Study procedures 20

8.4 Withdrawal of individual subjects 22
8.4.1 Specific criteria for withdrawal (if applicable) 22
8.5 Replacement of individual subjects after withdrawal 22
8.6 Follow-up of subjects withdrawn from treatment 22
8.7 Premature termination of the study 22
9. SAFETY REPORTING 22
9.1 Section 10 WMO event 22
9.2 AEs, SAEs and SUSARs 23
9.2.1 Adverse events (AEs) 23
9.2.2 Serious adverse events (SAEs) 23
9.2.3 Suspected unexpected serious adverse reactions (SUSARs) 23
9.3 Annual safety report 23
9.4 Follow-up of adverse events 24
9.5 Safety Committee 24
10. STATISTICAL ANALYSIS 24
10.1 Primary study parameter(s) 24
10.2 Secondary study parameter(s) 25
10.3 Other study parameters 25
10.4 Interim analysis (if applicable) 25
11. ETHICAL CONSIDERATIONS 25
11.1 Regulation statement 25
11.2 Recruitment and consent 25
11.3 Objection by minors or incapacitated subjects (if applicable) 25
11.4 Benefits and risks assessment, group relatedness 26
11.5 Compensation for injury 26
11.6 Incentives (if applicable) 26
12. ADMINISTRATIVE ASPECTS, MONITORING AND PUBLICATION 27
12.1 Handling and storage of data and documents 27
12.2 Monitoring and Quality Assurance 27
12.3 Amendments 27
12.4 Annual progress report 27
12.5 End of study report 27
12.6 Public disclosure and publication policy 27
12.7 Potential issues of concern 28
12.8 Synthesis 28
13. REFERENCES 29

LIST OF ABBREVIATIONS AND RELEVANT DEFINITIONS

**ABR**	**ABR form, General Assessment and Registration form, is the application form that is required for submission to the accredited Ethics Committee (In Dutch, ABR = Algemene Beoordeling en Registratie)**
**AE**	**Adverse Event**
**AR**	**Adverse Reaction**
**CCMO**	**Central Committee on Research Involving Human Subjects; in Dutch: Centrale Commissie Mensgebonden Onderzoek**
**CRF**	**Clinical record form**
**CV**	**Curriculum Vitae**
**DSMB**	**Data Safety Monitoring Board**
**GCP**	**Good Clinical Practice**
**IC**	**Informed Consent**
**IMPD**	**Investigational Medicinal Product Dossier**
**METC**	**Medical research ethics committee (MREC); in Dutch: medisch ethische toetsing commissie (METC)**
**(S)AE**	**(Serious) Adverse Event**
**Sponsor**	**The sponsor is the party that commissions the organisation or performance of the research, for example a pharmaceutical company, academic hospital, scientific organisation or investigator. A party that provides funding for a study but does not commission it is not regarded as the sponsor, but referred to as a subsidising party.**
**Wbp**	**Personal Data Protection Act (in Dutch: Wet Bescherming Persoonsgevens)**
**WMO**	**Medical Research Involving Human Subjects Act (in Dutch: Wet Medisch-wetenschappelijk Onderzoek met Mensen**

SUMMARY

Rationale: Community acquired pneumonia (CAP) is the largest cause of death in children and among the most frequent diagnoses in febrile children. We are in need to improve the recognition of children that benefit from antibiotic treatment for CAP.
Objective: To safely reduce antibiotic prescription by a clinical decision rule in febrile children suspected of CAP.
Study design: Stepped-wedge design with sequential implementation of a clinical decision rule guiding antibiotic treatment in children suspected of CAP at pediatric emergency departments of 8 participating hospitals.
Study population: Febrile children (1 month – 5 years) visiting the ED with signs of CAP
Intervention: *C*linical decision rule for the individual risk for CAP and for other SBI guiding a targeted approach for antibiotic prescription
Main study parameters/endpoints: *Primary:* Rate of antibiotic prescriptions and strategy failures; *Secondary:* Compliance to the rule; Duration/dosage of antibiotics; *Safety endpoints:* Number of complications of pneumonia, association of isolated pathogens with complicated CAP course.
Nature and extent of the burden and risks associated with participation, benefit and group relatedness: We plan to include a sample of 900 febrile children. This number is sensitive to detect a 15% reduction (low and intermediate risk) of antibiotic prescription rate in children suspected of CAP. The study acts during emergency visits initiated by the patient or his/her referrer, and collects data from patient history, physical examination and laboratory tests that are regularly occurring. The study does not result in prolonged visit or extra bloodsamples. The study will slightly increase patient burden by an extra control visit (telephone call in the majority, actual visit in about a quarter), as the indication of treatment is set more strict. Next, nasopharyngeal aspirates and swabs are collected for microbiological testing. Reduction of antibiotic prescription will reduce side-effects of antibiotics, that are regularly occurring, such as nausea and diarrhoea. The study may result in delayed antibiotic prescription and secondary hospitalisation. Severe complications of pneumonia are rarely occurring. The subject of the study (unnecessary antibiotic prescription in febrile children) is specific for young children as pneumonia is a major diagnosis in febrile children, and most children < 5 years suffer from viral causes.

1. INTRODUCTION AND RATIONALE

Community acquired pneumonia (CAP) is the single largest cause of death in children worldwide. In developed countries it exceeds the number of children, aged five and under, dying from meningitis and other infectious causes. CAP is among the most frequent diagnoses in children with fever. In developed countries CAP affects about 33 cases/10.000 children/year, with a hospital admission rate of 28 cases/10.000 children/year[1]. Given these numbers, there is general agreement that prompt and adequate therapy is essential to reduce the impact of the disease. Evidence-based antibiotic prescribing guidelines, however, have problems because there is no standard for establishing the diagnosis of CAP and controversies on type and duration of antibiotic prescription remain. We still observe a wide prescription of (often broad-spectrum) antibiotics for childhood CAP in practice [2, 3].

An estimated 30% (> 5 years) - 80% (< 2 years) of childhood CAP in western countries has viral causes [4, 5]. In the Netherlands *Streptococcus pneumoniae* and *Haemophilus influenzae* are the most common bacterial causes of CAP, sensitive to amoxicillin. Numerous studies have shown that chest radiography findings lack accuracy in defining the aetiology (viral, bacterial or atypical) of childhood pneumonia [6, 7]. Microbiological and viral testing on airway specimens and blood do not confirm or exclude bacterial causes, due to low sensitivity [9] and frequent concomitant occurrence of bacterial and viral causes [8, 9]. However, new insights suggest a relationship between nasopharyngeal microbiome and disease severity [10]. Clinical signs and symptoms and/or inflammatory tests have the same lack of complete accuracy. Next, the duration of antibiotic treatment for childhood CAP (current 7-10 days) is currently debated [5, 11]. As has been proved in adults before, a recent trial in childhood CAP showed non-inferiority of a 5-day treatment course compared to 7 or 10 days [12, 13]. A study on PCT guided treatment for childhood CAP could not prove a reduction of antibiotic prescriptions, concluding that additional clinical characteristics (i.e. prediction rules) may further contribute to define the indication for antibiotic treatment [14].

So in conclusion, we are in need to improve the recognition of those children that benefit from antibiotic treatment for bacterial causes of CAP. For adults, within the antibiotic stewardship programs to prevent antibiotic resistance from unnecessary use, the Dutch Working Party of Antibiotic Policy (SWAB) has proposed guidelines for antibiotic use in CAP, including a severity score for CAP [15]. These are, however, not applicable to children. We recently developed and broadly validated a prediction rule for children with fever that estimates the risk of pneumonia and of other serious bacterial infections for individual children (feverkidstool). The feverkidstool is based on clinical characteristics, vital signs and serum CRP [16]. The feverkidstool has passed all required phases to be implemented in routine clinical practice [17] and is available for practical use at the website of Erasmus MC (http://www.erasmusmc.nl/formulieren/feverkidstool). Impact analysis of this feverkidstool on diagnostic procedures in a selected population of 450 children with fever at the emergency department showed the feasibility of this approach and it's validity to estimate the risk for pneumonia and other serious infections (ZonMW 170992503). This study did not prove significant reduction on primary patient outcome (correct diagnosis), although reduction on some secondary outcomes (number of bloodtests). With respect to safety, the feverkidstool

advised to perform a chest radiograph in 42 children, of whom 25 had no CAP (60%, i.e. false positives). Routine care (controlgroup) resulted in 28 chest radiographs, including 16 children without CAP (57%). The feverkidstool advised to perform no chest radiograph in 178 children, of whom 2 had CAP afterwards (1%, false negatives), compared with 196 children without chest radiograph in routine care (controlgroup), including 1 child with CAP diagnosed afterwards (0.5%). Differences in false positive and false negative numbers in intervention and control group were not significant. We did not observe complications of delayed treatment of CAP in this trial of 450 children. In the study we observed 13% CAP among 236 children with fever and respiratory complaints; in contrast 39% was treated with antibiotics. As a next step we propose to use the feverkidstool for therapeutic guidance. As the feverkidstool discriminated well between bacterial and non-bacterial causes, we expect it will also be able to guide the indication for antibiotic prescription. In this study proposal we suggest an individualized therapeutic strategy for children suspected of CAP guided by the feverkidstool. Based on the predicted risk in the individual patient, the tool will advise either to withhold or to start of antibiotic treatment, and specifies antibiotic type and duration. This study will contribute to a reduction of antibiotic prescription in children with suspected CAP with unchanged outcome.

2. OBJECTIVES

The aim of this study is to reduce antibiotic prescriptions by use of a clinical decision rule (feverkidstool) in febrile children suspected for CAP with unchanged outcome.

Primary Objective:
Does the feverkidstool safely reduce unnecessary antibiotic prescriptions in children with CAP?

Secondary Objective(s)*:*
What is the compliance to the feverkidstool guiding clinicians on treatment for childhood CAP?

3. STUDY DESIGN

Cluster stepped wedge design with sequential implementation of the feverkidstool rule that advices on antibiotic treatment in febrile children suspected of community acquired pneumonia in participating hospitals over 8 time periods.

Duration: 24 months, centres will be randomized to implementation of the intervention within the period of 6-15 months after starting date of the study.

Setting: The study will be performed at 8 (mix of academic, teaching and non-teaching) hospitals in the South-West and central area of the Netherlands, with variable adherence, geographic and demographic areas, and ethnic population, i.e. Erasmus MC – Sophia, Sint Franciscus Hospital and Maasstad Hospital, Rotterdam, Reinier de Graaf Hospital, Delft,

Flevo-DKK Hospital, Almere, St. Elisabeth Hospital, Tilburg, LangeLand Hospital, Zoetermeer and the HAGA – Juliana Children's Hospital, Den Haag.

4. STUDY POPULATION

4.1 Population (base)

The targeted population are children aged 1 month – 5 years with fever (parent reported and/or measured during physical) with signs suspected of CAP. The study is limited to previously healthy children visiting the emergency department, and excludes febrile children with an obvious single other infectious focus (cutaneous, otitis media, rhinitis). Next, patients not understanding or not able to act on safety-net instructions in case of deterioration are excluded.

4.2 Inclusion criteria

In order to be eligible to participate in this study, a subject must meet all of the following criteria:
-Age 1 month – 5 years (maximum 60 months)
-Fever (parental reported elevated body temperature or measured during physical examination T > 38°C)
-Signs of lower respiratory tract infection (i.e. cough, dyspnea, tachypnea, rhales/wheezing or crepitations at auscultation).

4.3 Exclusion criteria

A potential subject who meets any of the following criteria will be excluded from participation in this study:
-Febrile children with an obvious single other infectious focus (cutaneous, otitis media, rhinitis)
-Medical comorbidity increasing the risk of infection or increasing the risk of complications to CAP (primary of secondary immunodeficiency, neurologic disease, cardiac, pulmonary disease)
-Patients with antibiotic use during the week prior to inclusion
-Patients with (self reported) allergy for amoxicillin
-Patiens with complicated pneumonia:

a) requiring intensive care (oxygen supply due to saturations <85%, or need of non-rebreathing mask or mechanical ventilation support)
b) pleuraempyema at first presentation
c) suspicion septicaemia/ hemodynamic instability

4.4 Sample size calculation

Sample size calculation is based on methods of Hussey and Hughes and the following assumptions [18]:
- 8 hospitals, starting at 7 moments, 8 study periods

- From previous studies (ZonMW 170992503), we expect 50% of children to be at low risk, 30% intermediate and 20% high risk, with a 23% (low risk), 35% (intermediate risk) and 85% (high risk) antibiotic prescription rate. We estimate to detect a reduction from 23% to 13% in the low risk population and a reduction of 35 to 20% in the intermediate risk population.

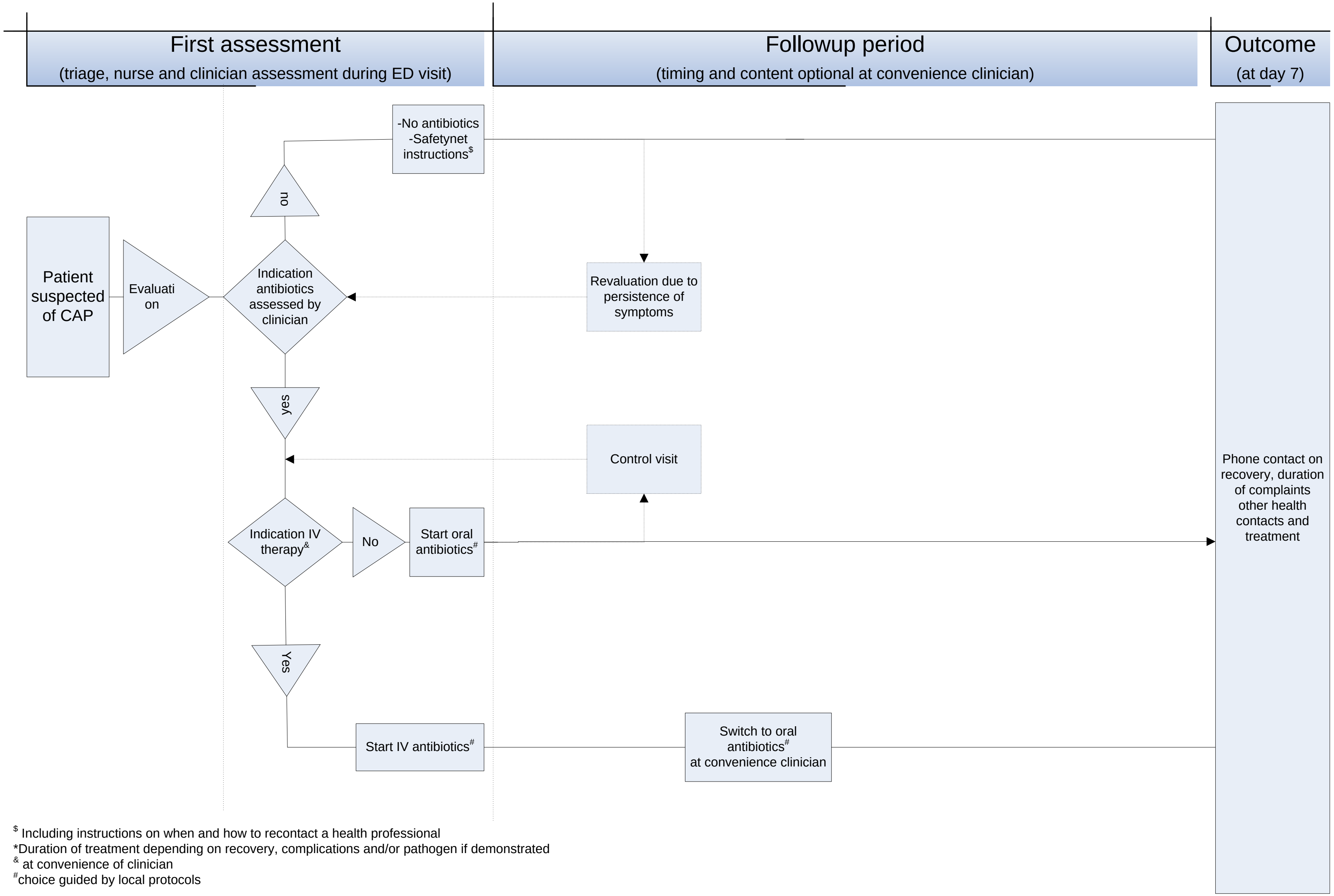

$ Including instructions on when and how to recontact a health professional
*Duration of treatment depending on recovery, complications and/or pathogen if demonstrated
& at convenience of clinician
#choice guided by local protocols

Figure 1. Flowchart study_preimplementationphase

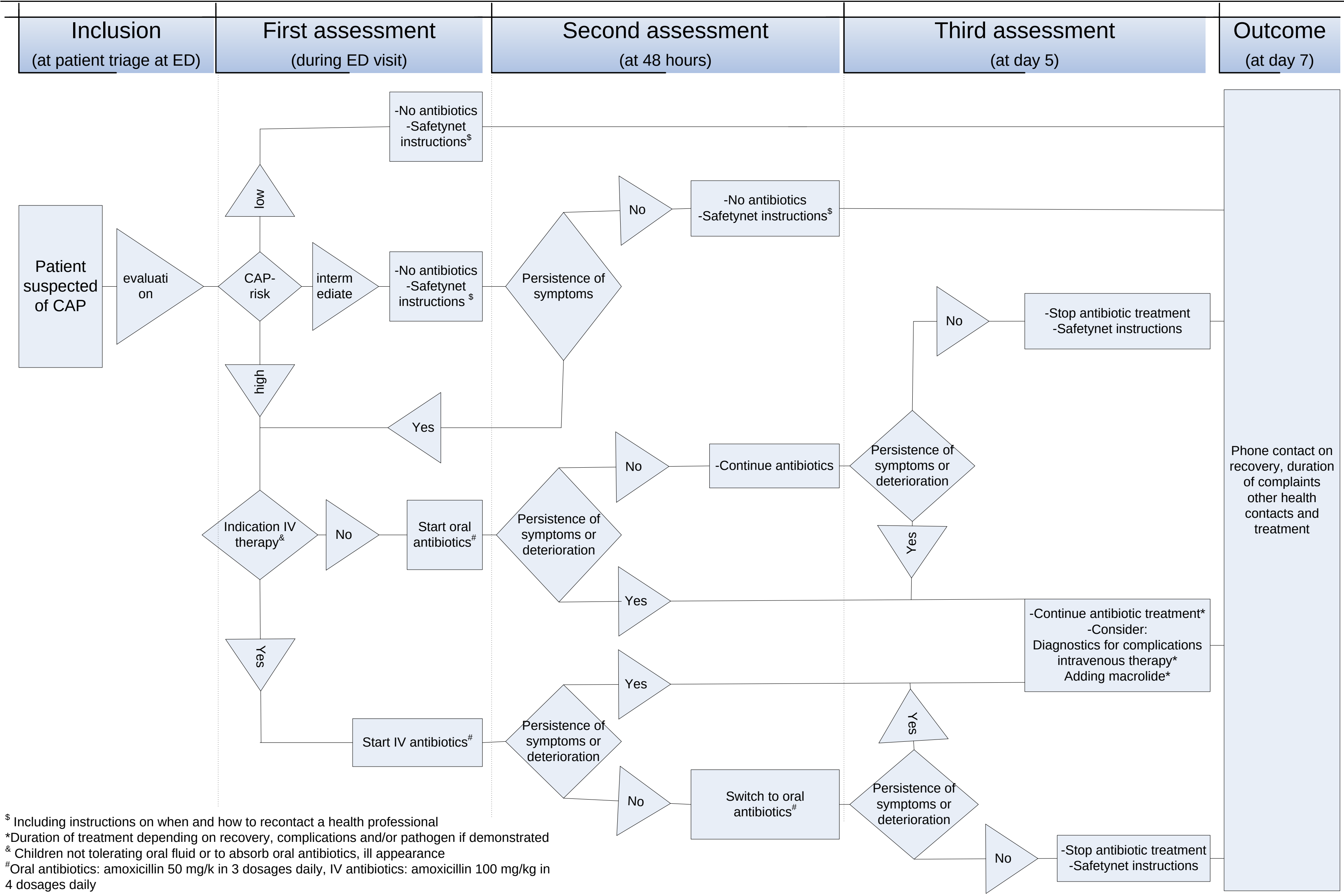

$ Including instructions on when and how to recontact a health professional
*Duration of treatment depending on recovery, complications and/or pathogen if demonstrated
& Children not tolerating oral fluid or to absorb oral antibiotics, ill appearance
#Oral antibiotics: amoxicillin 50 mg/k in 3 dosages daily, IV antibiotics: amoxicillin 100 mg/kg in 4 dosages daily

Figure 2. Flowchart study_postimplementation phase

-The intracluster correlatiecoefficient (ICC) is unknown, but we assume a power of 90% at independency will result in a power of 80% or more at multilevel analysis.

Modelling with these assumptions we estimate that a sample size of 1100 children with a suspicion of CAP in 24 months will be sensitive to detect a 10% (low risk) to 15% (intermediate risk) reduction of antibiotic prescription with a power of 0.9 and an alpha of 0.05. This number is also sensitive to detect a twofold increase of complications such as secondary hospitalisation or antibiotic treatment (currently being 5%). Additional severe complications as pleuraempyema will be registered.

Interim analysis of inclusions during the first year showed a higher antibiotic prescription than previously assumed: in low risk children we observed 35-45% antibiotic prescription; in the intermediate group this is 40%. Therefore we assume a reduction of 15% in both the low and intermediate risk group. Remodelling with these numbers and similar assumptions as above shows that an effect of 15-20% antibiotic reduction can be assessed in a sample of 800-900 children.

5. TREATMENT OF SUBJECTS

5.1 Investigational product/treatment

During the pre-implementation phase, children suspected of CAP are treated similar to current clinical practice. Figure 1 gives an impression of the possible steps, i.e. first assessment by the clinician who assess the indication for antibiotics. Followup, including possible control or revisits, is defined at the convenience of the treating clinician, and based on local protocols. This study additionally introduces a telephone call at day 7 after first ED visit to check for disease course and further health contacts and treatment.

During the post-implementation phase, we will introduce a clinical decision rule for febrile children, i.e. feverkidstool to advice on management (Figure 2). Based on clinical characteristics, vital signs, and serum CRP the individual risk for CAP and for other SBI will be estimated [16]. The feverkidstool includes clinical characteristics (age, gender, vital signs, description of general illness, fever duration and dyspnoea) and serum CRP. The feverkidstool provides a risk estimate for the diagnosis of CAP and will advise on antibiotic treatment based on defined risk thresholds. Additional information on the diagnostic performance of the feverkidstool and how the feverkidstool calculates the risk based on the presence or absence of clinical characteristics in the individual patient is described in figure 3.

We will develop a web based application of the feverkidstool (figure 4). The web page can be accessed from each desktop of participating hospitals. Further integration with the local available electronic hospital system is dependent on the technical possibilities of the different hospitals, but will be limited to the available budget. Integration aims to facilitate use of the feverkidstool during routine care.

Training ED personnel includes instruction of paediatricians (in training) on the clinical decision rule, knowledge in current guidelines on the diagnostic value of chest radiographs, and the risk of serious infections among different categories of children as defined by the clinical decision rule. The spread of knowledge on risks is one of main steps in reducing AB prescriptions. Personnel is instructed in giving safety net advice to parents, i.e. what signs indicate the need for seeking health care contact, and how they should do that. Reminders in the form of leaflets and posters will be present at the ED.

Computation feverkidstool risk estimate for pneumonia

FKTpneu= -17.888 + (age below 1 year*1.017) + (age above 1 year*-1.007) + (male sex*0.129) + (bodytemperature*0.286) + (durationfever (days)*0.213) + (presence of tachypnoea*0.436) + (presence of tachycardia*-0.043) + (presence of abnormal saturation*1.593) + (presence of impaired capillairy refill*-0.178) + (presence of breathing_difficulty*0.473) + (presence of ill appearance*0.162) + (value of lnCRP*0.639).
VARIABLE LABELS Logit_FKTpneu 'logit Feverkidstool (FKT) for pneumonia'.
EXECUTE.

Probability pneumonia (%)= EXP(FKTpneu)/ (1+EXP(FKTpneu)).
Lowrisk: Probability Pneumonia ≤ 3%
Intermediate risk: Probability Pneumonia 4-10%
High risk: Probability Pneumonia > 10%

Diagnostic performance

Receiver operating characteristic curve for the risk of pneumonia, and for the risk of other SBI

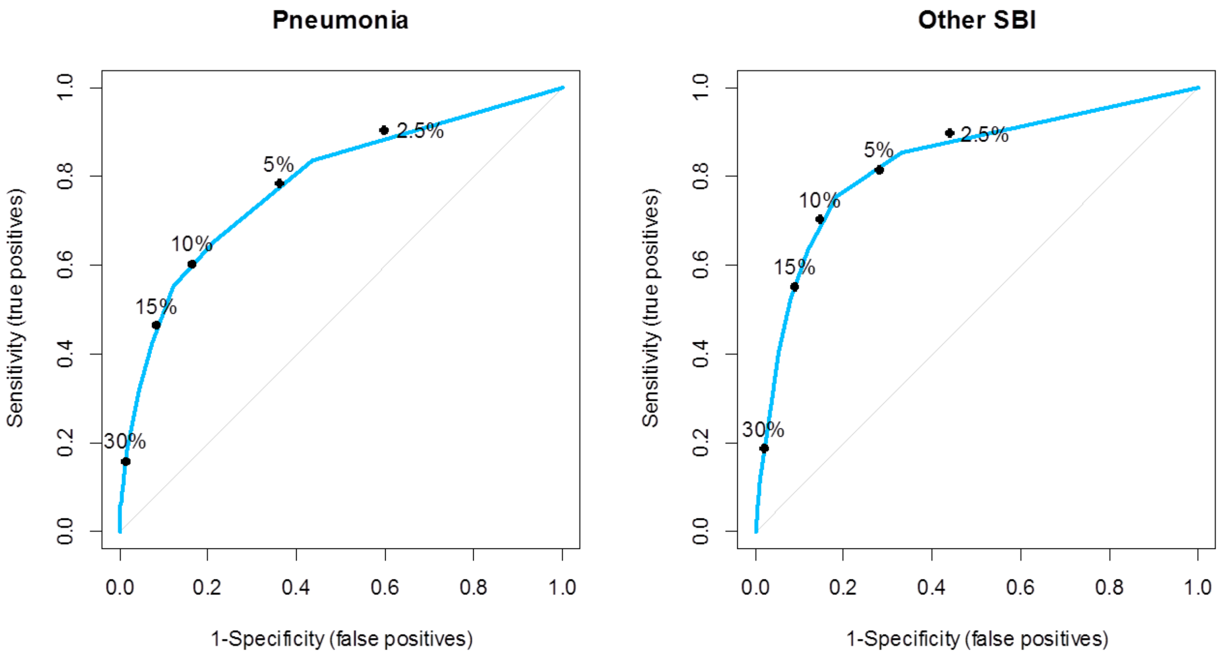

Figure 3. Description Feverkidstool and diagnostic performance

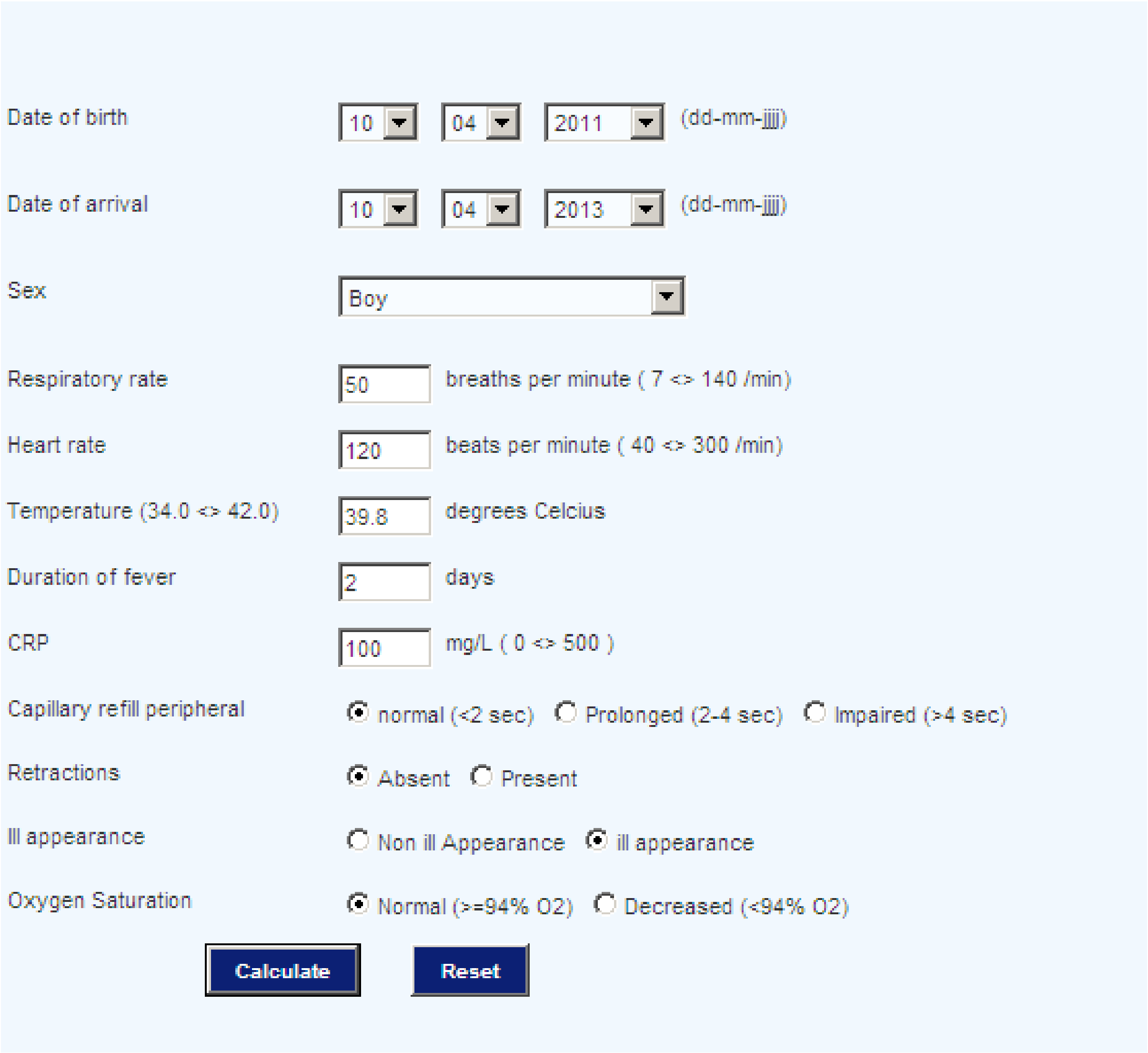

Figure 4. Visualisation of Feverkidstool on computerscreen

5.2 Use of co-intervention (if applicable)

NA

5.3 Escape medication (if applicable)

NA

6. INVESTIGATIONAL PRODUCT

NA

6.1 Investigational product/treatment

6.2 Name and description of investigational product(s)

6.3 Investigational product/treatment

6.4 Summary of findings from non-clinical studies

6.5 Summary of findings from clinical studies

6.6 Summary of known and potential risks and benefits

6.7 Description and justification of route of administration and dosage

6.8 Dosages, dosage modifications and method of administration

6.9 Preparation and labelling of Investigational Medicinal Product

6.10 Drug accountability

7. NON-INVESTIGATIONAL PRODUCT

NA

7.1 Name and description of non-investigational product(s)

7.2 Summary of findings from non-clinical studies

7.3 Summary of findings from clinical studies

7.4 Summary of known and potential risks and benefits

7.5 Description and justification of route of administration and dosage

7.6 Dosages, dosage modifications and method of administration

7.7 Preparation and labelling of Non Investigational Medicinal Product

7.8 Drug accountability

8. METHODS

8.1 Study parameters/endpoints

8.1.1 Main study parameter/endpoint

Number of narrow-spectrum antibiotic prescriptions: We will classify antibiotic prescriptions according to the Anatomical Therapeutic Chemical (ATC) classification system [19]. Drugs listed under J01CE (beta lactams sensitive penicillins) are considered the antibiotic of choice in CAP. We will compute the ratio between the number of broad spectrum penicillins, cephalosporins and macrolides and the number of narrow spectrum antibiotics.

Strategy failures: based on the clinical course during the 7 days after first visit, and includes children with persistence of respiratory distress (tachypnea, chest wall retractions, desaturation), or persistence of fever at the second or third assessment (48 hours and 5 days), secondary hospitalisation for CAP, need for change in therapy at second or third assessment (delayed antibiotic prescription, or intravenous treatment or switch of or adding antibiotics).This further includes children with complications related to pneumonia, such as pleura-empyema.

8.1.2 Other study parameters

Secondary study parameters/endpoints
Compliance to the feverkidstool: the number of children treated according to the feverkidstool versus the total number of children visiting the emergency department for CAP in the study period.

Safety endpoint:
Number of complications of pneumonia (pleurampyema, parapneumonic effusion, abscess, need for oxygen in days, respiratory insufficiency).
Association of isolated pathogens with complicated CAP course.
Descriptors of study sites, i.e. type (university, teaching, non-teaching), setting (inner-city, rural), adherence area, total number of children visiting the emergency care daily.

8.2 Randomisation, blinding and treatment allocation

NA

8.3 Study procedures

We will develop a web-based CRF including a database stored at a HTTPS certified web page. The webpage can be accessed from each desktop of participating hospitals. The CRF includes general patient characteristics, symptoms and signs, results of laboratory tests and/or chest radiographs, hospitalization, and data on treatment (antibiotic type,

dosing, duration) and disease course (information obtained during followup and at day 7). For the postimplementation phase, a web-based application of the clinical decision model (feverkidstool) is integrated in the CRF. Based on the feverkidstool an individual risk for pneumonia and for other SBI for each child is estimated that classifies the child into low, intermediate and high risk for pneumonia (Figure 3 and 4). During the post-implementation phase the webpage will include an advice to the clinician on the therapeutic strategy (see Figure 2):

-For low risk children (defined as a predicted risks of 3% or less), the strategy is conservative management, without additional testing or treatment, and instruction on deterioration and need for revisit.

-In intermediate risk children (defined as a predicted risk of 3 – 10%), the strategy includes watchful waiting management, without additional testing or treatment; children will be recontacted after 48 hours on persistence and/or deterioration of clinical symptoms and signs. In case of persistence of symptoms delayed antibiotic treatment is advised.

-In children with high risk predictions (predicted risk > 10%) the strategy is to initiate antibiotic treatment; pending on clinical signs treatment will be ambulatory or in hospital (see definitions) and intravenously or oral [1, 20]. Oral treated children will be re-evaluated after 48 hours for deterioration, which may be reason for further diagnostic evaluation of complications or change in therapy (intravenous or switch of/adding antibiotics). Intravenous treated children are re-evaluated after 48 hours, and in case of recovery, antibiotics will be continued orally [1]. All treated children will be evaluated at 120 hours. If recovery, antibiotics can be stopped [13]. Persistence or deterioration of symptoms may guide further diagnostic tests for complications of change in therapy.

All children (pre- and postimplementation phase) will be contacted after 7 days on uneventful recovery, potential other health care contacts and treatments. The antibiotic treatment included in the strategy is based on current guidelines [1, 20], that includes amoxicillin oral treatment 50 mg/kg/day in 3 dosages or intravenous treatment 100 mg/kg/day in 4 dosages.

According to current guidelines [1, 20], microbiologic testing is not routinely performed in children suspected of CAP. However, recent developments in the field show that specific patterns in the nasopharyngeal microbiome are associated with disease severity of LRTI[10]. To be able to explore infectious causes related to failures of therapy, and to increase insight in the relationship between nasopharyngeal colonisation and disease severity, we will collect samples for viral and bacterial testing in all included children. Nasopharyngeal aspirates are obtained for real time PCR for RSV, influenza A and B virus, hMPV and *Mycoplasma pneumoniae* [9], and stored for microbiome analysis. Samples will be frozen at -80°C and transported for analyzing as a batchd in the Erasmus MC Virology department. Purified nucleic acids will be used in real-time PCR assays for the detection of M. pneumoniae. Nasopharyngeal swabs are obtained for culture and amoxicillin susceptibility testing of potential bacterial respiratory pathogens *Streptococcus pneumoniae*, *Haemophilus influenzae*, *Staphylococcus aureus*, *Moraxella catarrhalis* and

Streptococcus pyogenes following standard microbiological procedures performed at the local hospital [21].

8.4 Withdrawal of individual subjects

Subjects can leave the study at any time for any reason if they wish to do so without any consequences. The investigator can decide to withdraw a subject from the study for urgent medical reasons.

As the study is merely implementation of evidence based medicine for the treatment of CAP, rather than a new intervention, withdrawal is not applicable. Consent is asked to use patient data for clinical research and to perform an extra professional health care contact.

To assess generalizability of the study results, we will compare included eligible children to non-included or withdrawed eligibles by age, gender, urgency (MTS category) and antibiotic prescription (yes/no). These data will be collected of all eligible patients and stored anomously.

8.4.1 Specific criteria for withdrawal (if applicable)

NA

8.5 Replacement of individual subjects after withdrawal

NA

8.6 Follow-up of subjects withdrawn from treatment

NA, all patients follow the same followup after visiting the ED.

8.7 Premature termination of the study

The study may potentially lead to increase of complications after pneumonia (see safety endpoint). Severe complications, like pleuraempyema are very rare occurring, and are monitored [14, 22]. A double increase of the severe complications during the post-implementation phase compare to the pre-implementation phase is reason to consider premature termination of the study.

9. SAFETY REPORTING

9.1 Section 10 WMO event

In accordance to section 10, subsection 1, of the WMO, the investigator will inform the subjects and the reviewing accredited METC if anything occurs, on the basis of which it appears that the disadvantages of participation may be significantly greater than was foreseen in the research proposal. The study will be suspended pending further review by the accredited METC, except insofar as suspension would jeopardise the subjects' health. The investigator will take care that all subjects are kept informed.

9.2 AEs, SAEs and SUSARs

9.2.1 Adverse events (AEs)

Adverse events are defined as any undesirable experience occurring to a subject during the study, whether or not considered related to the intervention. All adverse events reported spontaneously by the subject or observed by the investigator or his staff will be recorded.

9.2.2 Serious adverse events (SAEs)

AE not requiring immediate reporting during this study are: secondary hospitalisation and recurrent infection requiring treatment with antibiotics. These numbers are reported monthly and compared to the number during pre-implementation phase.

A serious adverse event is any untoward medical occurrence or effect that at any dose:

- results in death;
- is life threatening (at the time of the event);
- requires hospitalisation or prolongation of existing inpatients' hospitalisation;
- results in persistent or significant disability or incapacity;
- is a congenital anomaly or birth defect;
- Any other important medical event that may not result in death, be life threatening, or require hospitalization, may be considered a serious adverse experience when, based upon appropriate medical judgement, the event may jeopardize the subject or may require an intervention to prevent one of the outcomes listed above.

Local investigators will monitor severe adverse events (as. mentioned at severe complications, see safety endpoint paragraph 8.1.3) and report them to the coordinating investigator.
The sponsor will report the SAEs through the web portal *ToetsingOnline* to the accredited METC that approved the protocol, within 15 days after the sponsor has first knowledge of the serious adverse events.

SAEs that result in death or are life threatening should be reported expedited. The expedited reporting will occur not later than 7 days after the responsible investigator has first knowledge of the adverse event. This is for a preliminary report with another 8 days for completion of the report.

9.2.3 Suspected unexpected serious adverse reactions (SUSARs)

NA

9.3 Annual safety report

NA

9.4 Follow-up of adverse events

All AEs will be followed until they have abated, or until a stable situation has been reached. Depending on the event, follow up may require additional tests or medical procedures as indicated, and/or referral to the general physician or a medical specialist. SAEs need to be reported till end of study within the Netherlands, as defined in the protocol

9.5 Safety Committee

We have included a Data Safety Monitoring Board (DSMB), that constitutes of a clinical expert (paediatrician – pulmonologist), a methodologist and a representative for the patients (a member of the foundation Kind & Ziekenhuis). We will monitor the safety-endpoint (see paragraph 8.1.1) and compare them to numbers during pre-implementation phase. Cases of severe complications, such as pleuraempyema and need for invasive ventilation will be judged by the DSMB whether they are related to the applied protocol for antibiotic prescription.

10. STATISTICAL ANALYSIS

Primary, secondary and safety-endpoints during pre- and postimplementation phase will be presented by percentage with 95% confidence intervals. The population will be described in terms of age, gender, level of predicted risk of CPAP, clinical characteristics, triage urgency by percentage with 95% confidence intervals for categorical data or median (interquartile range) for continuous data.

The effect of the intervention will be estimated by comparing outcome measures in children in the post- with those in the pre-implementation phase. A generalized linear mixed model (GLMM) with antibiotic prescription as dependent will be used to correct for clustering in centers and time-effects. Time-effects will be included as fixed effect. Covariates includes the predicted risk for CAP (low, intermediate and high), patient age, triage urgency and season.

As the aim of the study is to estimate the effect of the intervention in routine practice, we will perform intention to treat analysis, i.e. children included in the post-implementation phase who did not follow the protocol, will be included in the analysis.

10.1 Primary study parameter(s)

Number of narrow-spectrum antibiotic prescriptions will be estimated and the ratio of narrow versus all antibiotic prescriptions (narrow and broad) will be computed by percentage with 95% confidence intervals (95% CI).
Number of strategy failures will be estimated by percentage with 95% confidence intervals.

10.2 Secondary study parameter(s)

The number of children that have been actually managed according to the protocol (figure paragraph 3) is expressed in a percentage (95% CI) of all eligible children.

10.3 Other study parameters

The frequency of complications of pneumonia (safety-endpoint) is expressed in a percentage (95% CI) of all included children.

We will perform an additional analysis to explore the association between identified viral pathogens in the nasopharyngeal aspirates and failures of therapy in children treated with amoxicillin, as well as the association between nasopharyngeal colonization with amoxicillin resistant respiratory bacterial pathogens and failures of therapy in children treated with amoxicillin. If the post-implementation shows an increased number of failures of therapy, one questions whether children with complications are comparable to those without from the microbiology perspective.

10.4 Interim analysis (if applicable)

Interim analysis is applied for safety endpoints, see paragraph 9.4 and 8.1.1

11. ETHICAL CONSIDERATIONS

11.1 Regulation statement

The study will be conducted according to the principles of the Declaration of Helsinki (version 64, date October 2013, Brazil) and in accordance with the Medical Research Involving Human Subjects Act (WMO).

11.2 Recruitment and consent

Subjects and their parents will be asked to participate in this study during their visit to the pediatric emergency care by the nurse. They will receive an information leaflet. Parents are asked on consent to use patient data for clinical research, and to perform a followup contact independent from routine medical care. In principle, parents are asked to complete the patient informed consent at the end of the emergency care visit, but may return the form by mail in the following week.

11.3 Objection by minors or incapacitated subjects (if applicable)

The study includes minors only, and parents of potential subjects are asked for consent. The study is considered as therapeutic study with potential benefit for the subject. Evaluation and examination at the first visit to the emergency care does not differ from usual care. From the study perspective we will introduce an extra professional health care contact, that most frequently can occur by telephone. We will obtain nasopharyngeal swabs and aspirates in children, that are considered common clinical practice.

11.4 Benefits and risks assessment, group relatedness

Parents usually fear the most serious outcome in febrile children, but are less aware of the actual risks of serious diagnoses and drawbacks of unnecessary treatment [23]. For the individual patient antibiotic use has risks of side-effects (nausea, diarrhea, allergic reactions). At population level it is associated with antibiotic resistance worldwide. The proposal implies pragmatic scientific research applied in routine care with direct benefit for the patient.

From the safety side, the study risks are considered to be acceptable for the patient. The strategy for initiating antibiotic treatment and on duration of therapy in this project is based on current guidelines and a well validated decision rule, and does not involve experimental approaches. Next, the study includes a well-defined follow-up scheme to detect potential complications and to initiate required. The burden to participate in this study concerns the very low risk of safety-endpoints, nasal pharyngeal aspirates and swabs for microbiological testing and one extra control visit, that may be performed by telephone in the majority.

The study has been discussed with the foundation Kind & Ziekenhuis, who recognize the relevance of the study. The principles of Kind & Ziekenhuis are optimizing the balance between the number of diagnostic and therapeutic procedures and the child’s quality of life; these fit to the present study.

11.5 Compensation for injury

The sponsor/investigator has a liability insurance which is in accordance with article 7, subsection 6 of the WMO.

The sponsor (also) has an insurance which is in accordance with the legal requirements in the Netherlands (Article 7 WMO and the Measure regarding Compulsory Insurance for Clinical Research in Humans of 23th June 2003). This insurance provides cover for damage to research subjects through injury or death caused by the study.

1. € 450.000,-- (i.e. four hundred and fifty thousand Euro) for death or injury for each subject who participates in the Research;
2. € 3.500.000,-- (i.e. three million five hundred thousand Euro) for death or injury for all subjects who participate in the Research;
3. € 5.000.000,-- (i.e. five million Euro) for the total damage incurred by the organisation for all damage disclosed by scientific research for the Sponsor as ‘verrichter’ in the meaning of said Act in each year of insurance coverage.

The insurance applies to the damage that becomes apparent during the study or within 4 years after the end of the study.

11.6 Incentives (if applicable)

NA

12. ADMINISTRATIVE ASPECTS, MONITORING AND PUBLICATION

12.1 Handling and storage of data and documents

The handling of personal data will comply with the Dutch Personal Data Protection Act. Data will be handled confidentially and anonymously. To be able to trace data to an individual subject, a subject identification code list will be used to link the data to the subject. In the anonymous database a research code is included for each individual. A key linking the research code to the patient identification number will be storage and safeguarded by the local investigators.

12.2 Monitoring and Quality Assurance

The pre-proposal has been positively judged by the National Health Council (ZonMW). The ethical committee of ErasmusMC has judged the project and agreed upon the monitoring plan. The study is considered to be of moderate risk.

12.3 Amendments

Amendments are changes made to the research after a favorable opinion by the accredited METC has been given. All amendments will be notified to the METC that gave a favorable opinion.

12.4 Annual progress report

The sponsor/investigator will submit a summary of the progress of the trial to the accredited METC once a year. Information will be provided on the date of inclusion of the first subject, numbers of subjects included and numbers of subjects that have completed the trial, serious adverse events/ serious adverse reactions, other problems, and amendments.

12.5 End of study report

The investigator will notify the accredited METC of the end of the study within a period of 8 weeks. The end of the study is defined as the last patient's last visit.

In case the study is ended prematurely, the investigator will notify the accredited METC within 15 days, including the reasons for the premature termination.

Within one year after the end of the study, the investigator/sponsor will submit a final study report with the results of the study, including any publications/abstracts of the study, to the accredited METC.

12.6 Public disclosure and publication policy

All results of this study will be disclosed unreservedly.

STRUCTURED RISK ANALYSIS

12.7 Potential issues of concern

NA

a. Level of knowledge about mechanism of action
b. Previous exposure of human beings with the test product(s) and/or products with a similar biological mechanism
c. Can the primary or secondary mechanism be induced in animals and/or in *ex-vivo* human cell material?
d. Selectivity of the mechanism to target tissue in animals and/or human beings
e. Analysis of potential effect
f. Pharmacokinetic considerations
g. Study population
h. Interaction with other products
i. Predictability of effect
j. Can effects be managed?

12.8 Synthesis

The strategy for initiating antibiotic treatment and on duration of therapy in this project is based on current guidelines and a well validated decision rule, and does not involve experimental approaches. Therefore, we skipped paragraph 12.7. The study proposal has been discussed with the foundation ‘Kind en Ziekenhuis (K&Z)’, who participates in the research group. They recognize the relevance of the study. Parents usually fear the most serious outcome in febrile children, but are less aware of the actual risks of serious diagnoses and drawbacks of unnecessary treatment [23]. For the individual patient antibiotic use has risks of side-effects (nausea, diarrhea, allergic reactions). At population level it is associated with antibiotic resistance worldwide. The proposal implies pragmatic scientific research applied in routine care with direct benefit for the patient. From the safety side, K&Z considers the study risks acceptable for the patient. The study includes a well-defined follow-up scheme to detect potential complications and to initiate required interventions. The study includes previously healthy children only, and excludes those who may be more susceptible to (complications of) delayed treatment of CAP because of underlying comorbidity. The study focus on children with the highest prevalence of viral causes of CAP that can safely be managed without antibiotics, i.e. children < 5 years. Main adverse events will be delayed antibiotic prescription in children with persistent complaints of fever and cough, and may therefore results in prolonged disease course. Severe complications of pneumonia, like pleuraempyema, are very rare, even in delayed treated patients.

13. REFERENCES

1. Harris, M., et al., *British Thoracic Society guidelines for the management of community acquired pneumonia in children: update 2011.* Thorax, 2011. **66 Suppl 2**: p. ii1-23.
2. Angoulvant, F., et al., *Impact of implementing French antibiotic guidelines for acute respiratory-tract infections in a paediatric emergency department, 2005-2009.* Eur J Clin Microbiol Infect Dis, 2012. **31**(7): p. 1295-303.
3. Otters, H.B., et al., *Trends in prescribing antibiotics for children in Dutch general practice.* J Antimicrob Chemother, 2004. **53**(2): p. 361-6.
4. Levy, M.L., et al., *Primary care summary of the British Thoracic Society Guidelines for the management of community acquired pneumonia in adults: 2009 update. Endorsed by the Royal College of General Practitioners and the Primary Care Respiratory Society UK.* Prim Care Respir J, 2010. **19**(1): p. 21-7.
5. Esposito, S., et al., *Antibiotic therapy for pediatric community-acquired pneumonia: do we know when, what and for how long to treat?* Pediatr Infect Dis J, 2012. **31**(6): p. e78-85.
6. Cherian, T., et al., *Standardized interpretation of paediatric chest radiographs for the diagnosis of pneumonia in epidemiological studies.* Bull World Health Organ, 2005. **83**(5): p. 353-9.
7. Lynch, T., et al., *A systematic review on the diagnosis of pediatric bacterial pneumonia: when gold is bronze.* PLoS One, 2010. **5**(8): p. e11989.
8. Doan, Q., et al., *Rapid viral diagnosis for acute febrile respiratory illness in children in the Emergency Department.* Cochrane Database Syst Rev, 2012. **5**: p. CD006452.
9. Spuesens, E.B., et al., *Carriage of Mycoplasma pneumoniae in the upper respiratory tract of symptomatic and asymptomatic children: an observational study.* PLoS Med, 2013. **10**(5): p. e1001444.
10. de Steenhuijsen Piters, W.A., et al., *Nasopharyngeal Microbiota, Host Transcriptome, and Disease Severity in Children with Respiratory Syncytial Virus Infection.* Am J Respir Crit Care Med, 2016. **194**(9): p. 1104-1115.
11. Haider, B.A., M.A. Saeed, and Z.A. Bhutta, *Short-course versus long-course antibiotic therapy for non-severe community-acquired pneumonia in children aged 2 months to 59 months.* Cochrane Database Syst Rev, 2008(2): p. CD005976.
12. Dimopoulos, G., et al., *Short- versus long-course antibacterial therapy for community-acquired pneumonia : a meta-analysis.* Drugs, 2008. **68**(13): p. 1841-54.
13. Greenberg, D., et al., *Short Course Antibiotic Treatment for Community-Acquired Alveolar Pneumonia in Ambulatory Children: A Double Blind, Randomized, Placebo Controlled Trial.* Pediatr Infect Dis J, 2014. **33**: p. 136-42.
14. Baer, G., et al., *Procalcitonin Guidance to Reduce Antibiotic Treatment of Lower Respiratory Tract Infection in Children and Adolescents (ProPAED): A Randomized Controlled Trial.* PLoS One, 2013. **8**(8): p. e68419.
15. Wiersinga, W.J., et al., *SWAB/NVALT (Dutch Working Party on Antibiotic Policy and Dutch Association of Chest Physicians) guidelines on the management of community-acquired pneumonia in adults.* Neth J Med, 2012. **70**(2): p. 90-101.
16. Nijman, R.G., et al., *Clinical prediction model to aid emergency doctors managing febrile children at risk of serious bacterial infections: diagnostic study.* BMJ, 2013. **BMJ 2013;346:f1706 doi: 10.1136/bmj.f1706**.
17. Reilly, B.M. and A.T. Evans, *Translating clinical research into clinical practice: impact of using prediction rules to make decisions.* Ann Intern Med, 2006. **144**(3): p. 201-9.
18. Hussey, M.A. and J.P. Hughes, *Design and analysis of stepped wedge cluster randomized trials.* Contemp Clin Trials, 2007. **28**(2): p. 182-91.
19. Methodology, W.C.C.f.D.S., *Guidelines for ATC classification and DDD assignment, 2013.* http://www.whocc.no. 2012: Oslo.
20. Bradley, J.S., et al., *The management of community-acquired pneumonia in infants and children older than 3 months of age: clinical practice guidelines by the Pediatric Infectious Diseases Society and the Infectious Diseases Society of America.* Clin Infect Dis, 2011. **53**(7): p. e25-76.
21. Versalovic, J., et al., *Manual of clinical microbiology, 10th ed.* 2011, Washington (District of Columbia): ASN Press.

22. Clark, J.E., et al., *Children with pneumonia: how do they present and how are they managed?* Arch Dis Child, 2007. **92**(5): p. 394-8.
23. Francis, N.A., et al., *Effect of using an interactive booklet about childhood respiratory tract infections in primary care consultations on reconsulting and antibiotic prescribing: a cluster randomised controlled trial.* BMJ, 2009. **339**: p. b2885.
