# Supplementary material for: Evaluation of a clinical decision rule to guide antibiotic prescription in children with suspected lower respiratory tract infection in The Netherlands: A stepped-wedge cluster randomised trial
Source: PLoS Med. 2020 Jan 31;17(1):e1003034. doi: 10.1371/journal.pmed.1003034 (PMC6993966; doi:10.1371/journal.pmed.1003034)
Supplement: S3 Text — (PDF) [file pmed.1003034.s008.pdf]

Assumptions power calculation

Original power calculation:

- 6 hospitals, 6 time periods
- Distribution risk groups: 50% low-risk, 30% intermediate-risk, 20% high-risk
- Antibiotic prescription at baseline: 23% in low-risk children, 35% in intermediate-risk children, 85% in high-risk children
- Effect of intervention on antibiotic prescription: 10%-point reduction in low-risk children, 15%-point reduction in intermediate-risk children, no difference in high-risk children
- Varying cluster sizes: 2 small, 2 medium, 2 large hospitals
- Seasonality: baseline inclusion rate, 2 months 50% higher rate, 2 months 50% lower rate
- ICC is unknown, assume 90% power to be sufficient

Interim power calculation:

- 6 hospitals, 6 time periods
- Distribution risk groups: 50% low-risk, 30% intermediate-risk, 20% high-risk
- Antibiotic prescription at baseline: 35-45% in low-risk children, 40% in intermediate-risk children, 85% in high-risk children
- Effect of intervention on antibiotic prescription: 10%-point reduction in low-risk children, 15%-point reduction in intermediate-risk children, no difference in high-risk children
- Varying cluster sizes: 2 small, 2 medium, 2 large hospitals
- Seasonality: baseline inclusion rate, 2 months 50% higher rate, 2 months 50% lower rate
- ICC is unknown, assume 90% power to be sufficient
