# Supplementary material for: Evaluation of a clinical decision rule to guide antibiotic prescription in children with suspected lower respiratory tract infection in The Netherlands: A stepped-wedge cluster randomised trial
Source: PLoS Med. 2020 Jan 31;17(1):e1003034. doi: 10.1371/journal.pmed.1003034 (PMC6993966; doi:10.1371/journal.pmed.1003034)
Supplement: S4 Text — (PDF) [file pmed.1003034.s009.pdf]

Statistical Analysis Plan

Protocol ID	Study to Reduce Antibiotic prescription in childhood Pneumonia: implementation of a validated decision rule
Short title	STRAP – Study to Reduce Antibiotic prescription in childhood Pneumonia
Version	0.5
Date	14 December 2018
Coordinating investigator/project leader	R. Oostenbrink, MD, PhD<br>r.oostenbrink@erasmusmc.nl
Principal investigator(s) per site	Erasmus MC: R. Oostenbrink, MD, PhD<br>Flevo ziekenhuis, Almere: A. van Wermeskerken, MD<br>Maasstad Ziekenhuis, Rotterdam: F. Smit, MD<br>Sint Franciscus Gasthuis, Rotterdam: G. Tramper-Stranders, MD, PhD<br>St. Elisabeth Ziekenhuis, Tilburg: C.C. Obihara, MD, PhD<br>Reinier de Graaf Gasthuis, Delft: J. G. Noordzij, MD, PhD<br>LangeLand Ziekenhuis, Zoetermeer: J. Punt, MD<br>Juliana Kinderziekenhuis, Den Haag: G.J.A. Driessen, MD, PhD
Statistician	Daan Nieboer
Author SAP	Josephine van de Maat, PhD Student

Contents

1. Introduction 2
2. Study Objectives and Endpoints 2
   Study objectives 2
   Endpoints 2
3. Summary of changes to the protocol 3
4. Study methods 3
5. Analysis and reporting 4
   a. Missing data 4
   b. Descriptive analyses 4
   c. Primary analysis: impact of Feverkidstool 5
      Analysis populations 5
      Outcome variables 5
      (1) Model: antibiotic prescription 5
      (2) Model: therapy failure 5
      (3) Safety endpoint: complications 6
   d. Secondary analyses 6
      Compliance 6
   e. Sensitivity analyses 6
      Effect of missing data 6
      Effect of period before and after roll-out phase 6
      Effect of not using CRP in pre-implementation phase 6
   f. Generalizability 6
References 7

1. Introduction

Community acquired pneumonia (CAP) is the single largest cause of death in children worldwide. In developed countries it exceeds the number of children, aged five and under, dying from meningitis and other infectious causes. CAP is among the most frequent diagnoses in children with fever. In developed countries CAP affects about 33 cases/10.000 children/year, with a hospital admission rate of 28 cases/10.000 children/year(1). Given these numbers, there is general agreement that prompt and adequate therapy is essential to reduce the impact of the disease. We are in need to improve the recognition of those children that benefit from antibiotic treatment for bacterial causes of CAP. We recently developed and broadly validated a prediction rule for children with fever that estimates the risk of pneumonia and of other serious bacterial infections for individual children (Feverkidstool). The Feverkidstool is based on clinical characteristics, vital signs and serum CRP (2). As the Feverkidstool discriminated well between bacterial and non-bacterial causes, we expect it will also be able to guide the indication for antibiotic prescription. In this study proposal we suggest an individualized therapeutic strategy for children suspected of CAP guided by the Feverkidstool. Based on the predicted risk in the individual patient, the tool will advise either to withhold or to start of antibiotic treatment, and specifies antibiotic type and duration. This study will contribute to a reduction of antibiotic prescription in children with suspected CAP with unchanged outcome.

These analyses will assess the efficacy and safety of Feverkidstool-guided therapy in comparison with routine care and will be included in the clinical study report.

2. Study Objectives and Endpoints

Study objectives

Primary Objective:

- Does the Feverkidstool safely reduce unnecessary antibiotic prescriptions in children with community acquired pneumonia?

Secondary Objective:

- What is the compliance of clinicians to the treatment advice of the Feverkidstool?

Endpoints

Primary endpoint

1. Number of antibiotic prescriptions at Emergency Department discharge.
2. Number of strategy failures.

Safety endpoint

- Number of complications of pneumonia

Secondary endpoint

- Number of children treated according to the Feverkidstool versus the total number of children included in the study during the intervention period.

3. Summary of changes to the protocol

Study methods and randomization (paragraph 4)
Original protocol: Duration: 24 months, centers will be randomized to implementation of the intervention within the period of 6-15 months after starting date of the study.
Change: Duration: 33 months, randomization to implementation of the intervention within 20 – 28 months after starting date of the study.

Data collection (paragraph 4, supplement 1, 2)
Original protocol: during post-implementation phase a third assessment of high-risk patients at day 5.
Change: this assessment was done at day 7.

Analyses, general (paragraph 8a)
Original protocol: only intention-to-treat analysis. Change: addition of per-protocol analysis.

Analyses, statistical model (paragraph 8c)
Original protocol: inclusion of covariates: predicted risk for CAP (low, intermediate and high), patient age, triage urgency and season. Change: adjustment of covariates.

4. Study methods

Design: Cluster stepped wedge design with sequential implementation of the Feverkidstool rule that advices on antibiotic treatment in febrile children suspected of community acquired pneumonia in participating hospitals over 7 time periods.

Setting: The study was performed at 8 (mix of academic, teaching and non-teaching) hospitals in the South-West and central area of the Netherlands, with variable adherence, geographic and demographic areas, and ethnic population, i.e. Erasmus MC – Sophia, Sint Franciscus Hospital and Maasstad Hospital, Rotterdam, Reinier de Graaf Hospital, Delft, Flevo-DKK Hospital, Almere, St. Elisabeth Hospital, Tilburg, LangeLand Hospital, Zoetermeer and the HAGA-Juliana Children's Hospital, Den Haag.

Intervention: Clinical decision rule (Feverkidstool) for the individual risk for CAP and for other serious bacterial infections guiding a targeted approach for antibiotic prescription. The Feverkidstool includes the following predictors: age, gender, duration of fever, ill appearance, retractions, capillary refill time, hypoxia (oxygen saturation <94%), tachypnea (based on APLS guideline), tachycardia (idem), temperature, CRP in mg/L.

Duration: January 2016 – September 2018 (33 months). January 2016 – August 2017 was the pre-implementation phase, in which routine care data were collected. At regular intervals (steps of 4 weeks) centres were randomized to start the implementation of the intervention between August 2017 and March 2018. This period was chosen to take the seasonality of respiratory tract infections into account. March 2018 – September 2018 was the post-implementation phase. Since 2 hospitals started in August 2017 with the pre-implementation phase due to logistical reasons, these hospitals were randomized for implementation of the intervention after period 3. The design is shown graphically in Figure 1.

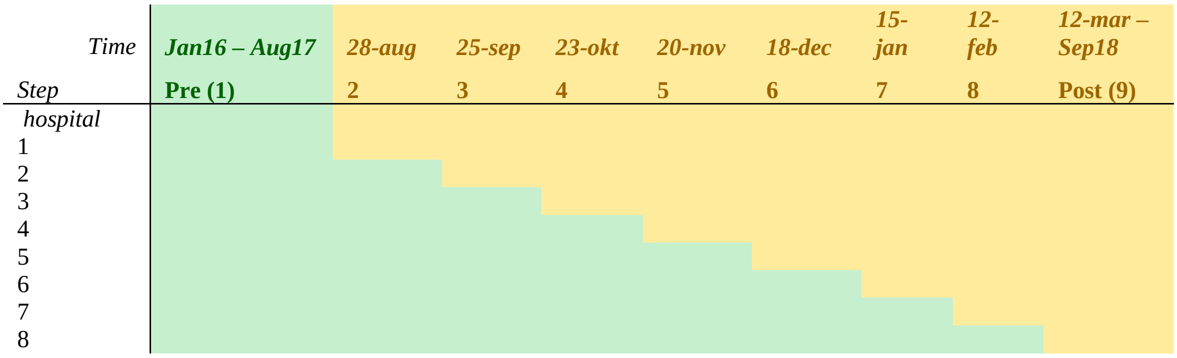

Figure 1. Design of STRAP

Data collection: The complete list of original collected variables is included as Appendix 1. In summary, the following variables were collected.

Variable	Inclusion at ED	Follow-up day 2 (during post-implementation phase)	Follow-up day 7
**General characteristics child**	X		
**History and examination**	X		
**CRP value**	X		
**X-ray**	X		
**Working diagnosis**	X		
**Antibiotic treatment**	X		
**Disposition**	X		
**General condition**		X	X
**Alarming signs**		X	
**Re-contact healthcare**		X	X
**Change of therapy**		X	X
**Secondary hospitalization**			X
**Duration of antibiotics**			X
**Oxygen therapy**			X

Some parents could not be reached in time for the 'follow-up day 7'. If the follow-up was performed later, all investigators were explicitly instructed to ask the parent about the situation of the child at day 7 after the ED visit. If follow-up was performed after >8 weeks only data will be used that could be verified from the patients electronic health record (e.g. duration of admission, oxygen therapy). Information about general condition of the child and presence of fever at day 7 will not be used given the risk of recall bias. Due to logistics of the participating hospitals, the third assessment of high-risk patients was taken together with the final follow-up at day 7 (see Appendix 2 and 3). During data collection all changes to the data were logged in the digital database Open Clinica.

Sample size: we estimated that a sample size of 900 children with a suspicion of CAP will be sensitive to detect 15 – 20% reduction of antibiotic prescription with a power of 0.9 and an alpha of 0.05. This number is also sensitive to detect a twofold increase of complications such as secondary hospitalization or antibiotic treatment (currently being 5%).

5. Analysis and reporting

The final analysis will be performed after the end of the trial, which is planned 30th of September 2018. The analyses will be performed on the clean datafile 'STRAP_final' and after finalization and approval of this SAP.

a. Missing data

Missing data will be assumed to be missing at random. Missing predictor variables in the models will be handled by multiple imputation using the mice package in R. The imputation model will include all variables needed for the analyses, and information on diagnosis, treatment, disposition and follow-up. Descriptive analyses will be performed on the non-imputed dataset, showing the amount of missing values per item.

b. Descriptive analyses

The population will be described in terms of age, gender, clinical characteristics, triage urgency by number (percentage) for categorical data or median (interquartile range) for continuous data. Primary and secondary endpoints during pre- and post-implementation phase will be presented by number (percentage). Number, dose, duration and type (first-line vs. second-line) of antibiotics prescribed at the ED will be presented.

Hospitals will be described in terms of hospital type (university, teaching, non-teaching), setting (inner city, regional), number of children visiting emergency care annually.

c. Primary analysis: impact of Feverkidstool

To analyse whether the Feverkidstool safely reduced unnecessary antibiotic prescriptions in children with CAP, we will perform two primary analyses to cover both the positive and negative effect of the : (1) the effect of the Feverkidstool on antibiotic prescription; and (2) the effect of the Feverkidstool on therapy failure. In addition, a separate safety endpoint will be reported.

Analysis populations

As the aim of the study is to estimate the unbiased effect of the intervention on antibiotic prescription, we will perform intention to treat analysis, i.e. the intervention group will be defined as all children included in the post-implementation phase, also those children in whom doctors did not adhere to the protocol.

In order to answer the clinical question what the impact of the Feverkidstool is in case of adherence to the protocol, we will also perform a per-protocol analysis of the primary outcome.(3) The intervention group will then be defined as all children in whom the Feverkidstool was calculated and who were treated according to the advice. Calculation of the Feverkidstool will be assumed when all Feverkidstool variables were recorded during ED visit. Since the per-protocol analysis is susceptible to confounding, randomisation will be used as an instrumental variable. The result of this is that children in the intervention phase in whom the protocol was not adhered to, will not be switched to the control group, but left out of the analysis. To minimize selection or confounding bias, we will adjust for factors that may influence participation in the study or adherence to the protocol.

For the second primary outcome 'therapy failure' analyses will be performed on all children with follow-up information on therapy failure available.

Outcome variables

Variable	Definition
**Antibiotic prescription**	Antibiotics prescribed at ED discharge (yes/no)
**Therapy failure**	Based on follow-up:<br>Need for supplemental oxygen up to day 7<br>*OR* persistence of fever up to day 7<br>*OR* hospitalization for CAP after initial discharge<br>*OR* delayed antibiotics<br>*OR* switch to iv antibiotics<br>*OR* switch of antibiotics<br>*OR* adding of antibiotics<br>*OR* complications of pneumonia (pleura-empyema, abscess, respiratory insufficiency)

(1) Model: antibiotic prescription

In the primary analysis a multilevel generalized linear mixed model will be used to calculate the impact of the intervention on antibiotic prescription.

The unadjusted model will be: *main_model = glmer (antibiotics ~ (1|hospital) + step + intervention)*

In which *antibiotics* is antibiotic prescription at ED discharge (yes/no), *step* is the period of stepwise implementation (1 – 9) and *intervention* is care according to Feverkidstool (before / after implementation). *Hospital* is added as a random effect, to take clustering at hospital level into account.

We will also fit a model where we adjust for potential confounders. We will include the following general or clinically relevant confounders: age, gender, season, duration of fever and ill appearance.

adjusted_model = glmer (antibiotics ~ (1|hospital) + step + intervention + age + gender + season + duration of fever + ill appearance

(2) Model: therapy failure

To calculate the impact of the intervention on therapy failure a similar multilevel generalized linear mixed model will be used:

main_failure = glmer (failures ~ (1|hospital) + step + intervention)

adjusted_failure = glmer (failures ~ (1|hospital) + step + intervention + age + gender + season + duration of fever + ill appearance

(3) Safety endpoint: complications

The number of complications of pneumonia, adverse events and serious adverse events will be reported.

Variable	Definition
**Complications**	Presence of pleura-empyema OR parapneumonic effusion OR abscess OR respiratory insufficiency (defined as need for invasive mechanical ventilation)
**Adverse Event (AE)**	Secondary hospitalization OR recurrent infection requiring treatment with antibiotics
**Serious Adverse Event (SAE)**	Death OR life-threatening event OR persistent or significant disability

d. Secondary analyses

Compliance

The level of compliance will be described as the number (and percentage) of children that are treated according to the Feverkidstool, out of the total number of children included in the study during the intervention period. We will report the level of compliance throughout the study and for different hospitals.

Variable	Definition
**Compliance to Feverkidstool**	All Feverkidstool variables available AND patient treated according to advice (e.g. no antibiotics prescribed in child with low/intermediate risk)

e. Sensitivity analyses

Effect of missing data

To evaluate the effect of the imputation of missing predictor variables on our primary outcome, we will perform a sensitivity analysis on all predictors of which >10% was missing in the dataset. The change in effect will be evaluated by: 1) complete case analysis (removing cases with that missing predictor); 2) analysis simulating missing = low (for continuous predictors) or absent (for dichotomous predictors; 3) analysis simulating missing = high or present.

To evaluate the effect of loss-to-follow-up on the outcome therapy failure, we will perform a sensitivity analysis under the assumption of: 1) missing follow-up = no therapy failure, and 2) missing follow-up = therapy failure.

Effect of period before and after roll-out phase

To evaluate the effect of the prolonged period before the roll-out phase (January 2016 – July 2017) and after the roll-out phase (May – September 2018) we will perform a sensitivity analysis of the primary outcome, only using data from the roll-out period. This is defined as 4 weeks before hospital 1 started the intervention, until 4 weeks after the last hospital started the intervention.

Effect of not using CRP in pre-implementation phase

In two hospitals CRP was not routinely performed in the pre-implementation phase, but started only in the intervention phase. To adjust for this factor, we will perform the main analyses on the other 6 hospitals who did perform routine CRP measurement throughout both phases of the study.

f. Generalizability

In order to evaluate the generalizability of our findings, we will compare the patients included in our study to the patients that were not included in our study during the study period, in terms of age, triage urgency, number of antibiotic prescriptions and number of hospitalizations.

References

1. Harris M, Clark J, Coote N, Fletcher P, Harnden A, McKean M, et al. British Thoracic Society guidelines for the management of community acquired pneumonia in children: update 2011. Thorax. 2011;66 Suppl 2:ii1-23.
2. Nijman RG, Vergouwe Y, Thompson M, Van Veen M, Van Meurs AH, Van der Lei J, et al. Clinical prediction model to aid emergency doctors managing febrile children at risk of serious bacterial infections: diagnostic study. BMJ. 2013;BMJ 2013;346:f1706 doi: 10.1136/bmj.f1706.
3. Hernan MA, Robins JM. Per-Protocol Analyses of Pragmatic Trials. N Engl J Med. 2017;377(14):1391-8.
