# Supplementary material for: Evaluation of a clinical decision rule to guide antibiotic prescription in children with suspected lower respiratory tract infection in The Netherlands: A stepped-wedge cluster randomised trial
Source: PLoS Med. 2020 Jan 31;17(1):e1003034. doi: 10.1371/journal.pmed.1003034 (PMC6993966; doi:10.1371/journal.pmed.1003034)
Supplement: Attachment — Submitted filename: Response to reviewers_181119.docx [file pmed.1003034.s010.docx]

Manuscript reference number: PMEDICINE-D-19-03060

Title: A clinical decision rule guiding antibiotic prescription for childhood pneumonia: a stepped-wedge, cluster randomized trial

Dear Clare Stone,

Thank you for reviewing our paper entitled: "A clinical decision rule guiding antibiotic prescription for childhood pneumonia: a stepped-wedge, cluster randomized trial". We are very pleased to hear that our manuscript is considered for publication in PLOS Medicine and we have taken this opportunity to clarify some issues raised by the reviewers. We thank the reviewers for their valuable comments to improve the paper, and have revised our manuscript according to their recommendations.

Foremost, the reviewers acknowledge the importance of our trial to evaluate an intervention to safely reduce antibiotic prescriptions. They mentioned that although the decision rule did not achieve an overall reduction in antibiotic prescriptions, it appears the decision rule has benefits in both reduction and strategy failures. The main concerns of the reviewers were related to the sample size calculation in our study, and thereby the power of our conclusions. We regret that this raised confusion, and have provided detailed explanations to the reviewers’ specific questions and have clarified this throughout the revised manuscript. We have added the exact assumptions of the power calculation as a supplementary file S3. Moreover, we have repeated the sample size calculations as a post-hoc sensitivity analysis, accounting for clustering at different ICCs and for multiple testing. This showed that our study was sufficiently powered, also after adjusting for multiple testing because of two co-primary outcomes. This was confirmed by the calculation of 97.5% confidence intervals of the primary analyses, as requested by reviewer #1, comment 2. We have provided the details of the post-hoc (sensitivity) sample size calculation in the rebuttal below. We have not included these results in the revised manuscript, because it was not described in the original approved protocol and predefined analysis plan. However, if the editors or reviewers think it is necessary, we could include the following comment in the Discussion section, page 22, line 452:

*“Post-hoc sensitivity analysis of the sample size calculation showed that our study was sufficiently powered (power of 0.8), also when accounting for clustering at varying ICC and adjusted for multiple testing.”*

Otherwise, the reviewers mainly asked for more clarification. We feel we were able to address all concerns in the revised version; our response to the individual review items can be found below.

Kind regards, on behalf of all co-authors,

Josephine van de Maat

Erasmus MC – Sophia Children’s Hospital

Rotterdam, The Netherlands

Dr. R. Oostenbrink

Paediatrician – epidemiologist

Requests from the editors:

**Comment 1.** Title – please add country. **Answer 1:** Done

**Comment 2**. Abstract, please add some summary demographic information on the children in the study. Please **add p values where 95%Cis are given (also in main text and tables).** Please add a sentence on the study’s limitations as the final sentence of the ‘Methods and findings’ section of the abstract.

**Answer 2.** We have added p-values at all points where confidence intervals were presented, in text and tables. And we have added more demographic information and limitations in the abstract, page 3, line 65 and 71:

*“Most children (77%) were referred by a general practitioner and half of children were hospitalized.”*

And:

*“Main limitations of the study were missing CRP-values in the pre-intervention phase and a prolonged baseline period due to logistical issues.”*

**Comment 3**. Data – data needs to be available (even if through request for restricted data sets because of ethical restrictions) from publication to comply with PLOS data policy. Note also that an author cannot be a point of contact to request access. PLOS Medicine requires that the de-identified data underlying the specific results in a published article be made available, without restrictions on access, in a public repository or as Supporting Information at the time of article publication, provided it is legal and ethical to do so.

**Answer 3.** The underlying data of this article have been deposited in the repository of Data Archiving and Networked Services (DANS, https://doi.org/10.17026/dans-27a-fj4k). We have changed the point of contact from an author to the contact person of DANS (info@dans.knaw.nl).

**Comment 4.** At this stage, we ask that you include a short, non-technical Author Summary of your research to make findings accessible to a wide audience that includes both scientists and non-scientists. The Author Summary should immediately follow the Abstract in your revised manuscript. This text is subject to editorial change and should be distinct from the scientific abstract. Please see our author guidelines for more information:

**Answer 4.** We have added a non-technical Author Summary.

**Comment 5.** Refs need to be in square brackets in the main text and please use the "Vancouver" style for reference formatting, and see our website for other reference guidelines

**Answer 5.** We used Vancouver style and square brackets for referencing.

**Comment 6.** The start date for the trial / recruitment in your paper do not match the dates at the trial registration page provided. Please comment. Also there is a discrepancy between the number of registrants needed and how many were recruited (1100 / 900). Please provide more details (line 128) and also comment on whether the study is underpowered.

**Answer 6.** The trial register (www.trialregister.nl/trial/5178) mentions a start date of 1 October 2015. This was the anticipated start date when the trial was registered and ethical clearance was obtained. Due to logistics of setting up the data collection in different hospitals, the actual recruitment of patients started 3 months later, in January 2016. The trial register indeed mentions 1100 inclusions as a target size. However, under the heading ‘Summary’ it also describes the interim analysis that was performed after the first year of data collection. This was done before introduction of the intervention, so it was blinded to the outcome of the trial.[1] A substantially higher than anticipated antibiotic prescription rate in the low-intermediate and high risk group was observed. Therefore we redid the sample size calculation using these observed prescription rates, showing that a sample size of 800-900 was sufficient to test our hypothesis. We have made this more explicit in the revised Methods section.

Methods, page 6, line 161:

*“(…) the sample size was adjusted from 1100 to 900 children, which is also reported in the NTR.”*

Methods, page 10, line 258 and 264:

*“Interim analysis of inclusions during the first year showed a higher baseline prescription than was assumed. An interim power calculation based on this assumption (…)”*

*“The interim power analysis was performed before introduction of the intervention, so it was blinded to the outcomes of the trial.”*

Since we reached a sample size of 999 children, our study is sufficiently powered. We added this comment in the ‘Strengths and limitations’ part of the Discussion (page 21, line 423):

*“We had complete information on the outcome antibiotic prescription, good compliance to the protocol, a high follow-up rate and sufficient power”*

**Comment 7.** Line 451 – please confirm consent was written. **Answer:** done

**Comment 8.** Please provide a CONSORT checklist as a supp file, using sections and paragraph numbers instead of pages. Apologies if I have missed this. **Answer:** done.

**Comment 9.** Please remove the data statement text from the main Word doc – it is automatically pulled in from the submission form. **Answer**: Done.

**Comments from the reviewers:**

**Reviewer #1:** This articles reports the results of a stepped-wedge trial assessing the impact of an intervention aimed at reducing antibiotics prescription in low-risk children with pneumonia. The trial includes 8 hospitals, sequentially randomised to the intervention. I have listed my comments below:

**Comment 1**. I was not able to visualise Figure S1 which was provided in .eps format

**Answer 1:** We are sorry to hear, but .eps was the requested format for figures. In the revised manuscript this figure is added to the main text as Figure 1, so it is visible now within the manuscript as well.

**Comment 2.** Sample size calculation (cf Lines 197-198): Please provide justification for not adjusting for multiple testing in the presence of two co-primary outcomes. I would also like to understand whether the significant results seen for the strategy failure outcome remain significant using a significance threshold of 2.5% (and a 97.5% confidence interval).

**Answer 2:** At the time of sample size calculation it was not deemed to be necessary to adjust for multiple testing, since both outcomes were considered interdependent, although this can be questioned from a statistical point of view. Following the reviewers remark, we have repeated the power calculation as a sensitivity analysis, adjusting for multiple testing, accounting for clustering (at different ICCs) and including 8 hospitals. This showed that we achieved sufficient power (power 0.8) with our sample size of 999 children. Figure 1 below shows the sensitivity analyses of the power calculations: a = original analysis sample size, b = interim analysis sample size.

*Figure 1a. Sensitivity analysis sample size calculation, using varying ICC, significance level 5% and 2.5%.*

*Figure 1b. Interim sample size calculation, using observed prescription rates from first year, varying ICC*

We have not included these results in the revised manuscript, because it was not described in the original approved protocol and analysis plan. If the reviewer thinks it is necessary, we could include the following comment in the Discussion section, page 22, line 452:

*“Post-hoc sensitivity analysis of the sample size calculation showed that our study was sufficiently powered (power of 0.8), also when accounting for clustering at varying ICC and adjusted for multiple testing.”*

Moreover, we have calculated the 97.5% confidence intervals for all primary analyses:

|  | **Pre-intervention** | **Intervention** |  |  |  |  |
| --- | --- | --- | --- | --- | --- | --- |
| **Primary analyses** |  |  | Unadjusted OR (95% CI) | Unadjusted OR (97.5% CI) | Adjusted OR (95% CI) | Adjusted OR (97.5% CI) |
| Intention to treat population |  |  |  |  |  |  |
| - Antibiotic prescription | 179/597 (30%) | 101/402 (25%) | 1.06 (0.61-1.85) | 1,06 (0,56-2) | 1.07 (0.57-2.01) | 1,07 (0,52-2,2) |
| - Strategy failure | 131/572 (23%) | 61/381 (16%) | 0.56 (0.34-0.93) | 0,56 (0,32-1) | 0.53 (0.32-0.88) | 0,53 (0,3-0,94) |
|  |  |  |  |  |  |  |
| Per protocol population |  |  |  |  |  |  |
| - Antibiotic prescription | 179/597 (30%) | 83/359 (23%) | 0.89 (0.5-1.61) | 0,89 (0,46-1,75) | 0.96 (0.49-1.88) | 0,96 (0,45-2,07) |
| - Strategy failure | 131/572 (23%) | 57/340 (17%) | 0.6 (0.36-1) | 0,6 (0,33-1,07) | 0.56 (0.34-0.93) | 0,56 (0,32-1) |
|  |  |  |  |  |  |  |
|  |  |  |  |  |  |  |

We assumed that the request for 97.5% CI was for your own information, and because it was not included in the original approved protocol we have not added this to the revised manuscript. All strategy failure outcomes (except the unadjusted per-protocol) remain significant at this lower significance threshold.

**Comment 3.** Please provide the assumptions made behind the statement "The intra cluster correlation coefficient was unknown, but we assumed a power of 90% at independency would result in a power of 80% or more at multilevel analysis." Based on my experience, a loss of only 10% power between an independent and a cluster (stepped-wedge) trial seems somewhat unlikely. I understand that the ICC was unknown but I would like the authors to tell us what ICC was compatible with this minimal loss of power. In addition, given the observed ICC of 0.04, I would like to understand what power was attainable with the sample size and for the targeted difference.

**Answer 3.** To answer the question of the reviewer, we refer to the Figure 1 of the previous comment. We repeated the power calculations (as a sensitivity analysis), accounting for clustering, and simulating varying the ICC. All show that our trial had sufficient power in detecting the hypothesized treatment effect. For transparency to the reader we explained in more details in the methods the assumptions on which the original and interim sample size calculations were based and we listed these assumptions in a Supplementary file S3.

**Comment 4.** There appears to be an inconsistency in the OR calculation for Antibiotic prescription. According to my raw calculation 101/402 vs 179/597 leads to an OR of 0.78 in favour of the intervention (or 1.28 using the intervention as the reference and not 1.06 as reported in Table 2). I understand that the unadjusted OR is in fact adjusted for the time period and that the OR estimated from the model is likely to be different from the one obtained via a simple calculation like mine; however, this seems like a big difference. Please confirm.

**Answer 4:** We thank the reviewer for this question. It is indeed confusing to see a raw difference in favour of the intervention, while an OR of >1 is reported. The ORs are not reported in an inconsistent way, but the analysis underlying the computation of the ORs make them differ from the simple calculation. First, the ORs are adjusted for time period, as the reviewer already mentions. Second, the inclusion of ‘hospital’ as a random effect in the multilevel model, to account for clustering at hospital level, affects the estimates. Moreover, the raw calculation is also affected by the between-hospital and between-phases differences. In our study we had high and low-prescribing hospitals; if a low-prescribing hospital switches relatively late to the intervention in the trial, they contributed more to the pre-intervention arm, so it would seem that the pre-intervention group has a low prescription rate compared to the intervention group. However, the intervention may still decrease the prescription rate in each of the individual hospitals leading to an OR below 1 in the analysis of the data. To improve clarity on this point, we have added a supplementary table (S7) showing the exact prescriptions per hospital and per time-period, as the reviewer suggested in comment 6.

**Comment 5.** The odds ratios for antibiotic prescription and strategy failure (both 'bad' outcomes) appear to be computed in different directions, i.e. once using the intervention period as the reference and once using the pre-intervention period as the reference. This is confusing and I would suggest using the pre-intervention as the reference for all estimates so that an odds ratio smaller than 1 always corresponds to an improvement with the intervention.

**Answer 5:** This relates to question and answer 4. We confirm that all odds ratios are computed in the same direction, taking the pre-intervention period as the reference. The confusing OR is the one for antibiotic prescription, that is >1. We refer to answer 4 for the explanation of that OR.

**Comment 6.** Please consider adding a figure showing the proportion of patients with antibiotic prescription for each period within each hospital so we can visualise the patterns over time.

**Answer 6.** We thank the reviewer for this suggestion and have added a table with this information as a supplementary file. We provided the crude numbers, instead of the pattern of proportions. It was not helpful to visualize the pattern of proportions, because a categorization per hospital and per time period resulted in low numbers per cell (hence high variability in proportions). Therefore we have not added this to Figure 1 in the main body, but as a supplementary file S7:

**Supplementary file 7. Antibiotic prescription per hospital and per time period**

| **Time period** | **1** | **2** | **3** | **4** | **5** | **6** | **7** | **8** | **9** |
| --- | --- | --- | --- | --- | --- | --- | --- | --- | --- |
| **Hospital** | n/N (%) | n/N (%) | n/N (%) | n/N (%) | n/N (%) | n/N (%) | n/N (%) | n/N (%) | n/N (%) |
| **A** | 11/69 (16%) | 0/7 (0%) | 2/9 (22%) | 2/12 (17%) | 5/28 (18%) | 5/15 (33%) | 4/20 (5%) | 5/17 (29%) | 2/19 (11%) |
| **B** | 8/33 (24%) | 0/2 (0%) | 1/3 (33%) | 0/6 (0%) | 1/16 (6%) | 2/15 (13%) | 1/3 (33%) | 1/1 (100%) | 3/6 (50%) |
| **C** | 10/27 (37%) | 0/1 (0%) | 1/1 (100%) | 1/5 (20%) | 0/3 (0%) | 0/1 (0%) | 2/3 (67%) | 1/2 (50%) | 2/3 (67%) |
| **D** | 35/74 (47%) | 3/6 (50%) | 1/8 (13%) | 3/7 (43%) | 6/23 (26%) | 3/9 (33%) | 0/4 (0%) | 3/5 (60%) | 11/28 (39%) |
| **E** | 0/0 (0%) | 0/0 (0%) | 1/5 (20%) | 0/3 (0%) | 3/12 (25%) | 0/2 (0%) | 0/3 (0%) | 0/1 (0%) | 2/5 (40%) |
| **F** | 23/52 (44%) | 0/0 (0%) | 1/4 (25%) | 4/8 (50%) | 0/9 (0%) | 2/9 (22%) | 2/5 (40%) | 0/0 (0%) | 5/11 (45%) |
| **G** | 1/1 (100%) | 3/17 (18%) | 1/12 (8%) | 5/23 (22%) | 19/51 (37%) | 6/20 (30%) | 5/20 (25%) | 5/10 (50%) | 18/57 (32%) |
| **H** | 27/71 (38%) | 0/3 (0%) | 0/2 (0%) | 1/6 (17%) | 1/10 (10%) | 2/11 (18%) | 1/13 (8%) | 1/7 (14%) | 6/45 (13%) |

**Comment 7.** Please provide the ICC for both co-primary endpoints in Table 2.

**Answer 7.** For the primary endpoint therapy failure no clustering at hospital level was observed in our study, so the observed ICC is 0. We have added this to Table 2.

**Comment 8.** Please add p-values to Table 2 for at least the primary and secondary analyses.

**Answer 8.** We have added the p-values.

**Comment 9.** In Table 2, please add a note to explain what the sensitivity analysis labelled "population around roll-out period" consist of.

**Answer 9.** This was indeed only mentioned in the Methods section. We have now added this information as a footnote to Table 2:

*“‡ Using data from four weeks before until four weeks after the roll-out period, resulting in nine time-periods of equal length, accounting for the prolonged baseline and post roll-out period.”*

**Comment 10.** Looking at the different components of the "strategy failure" outcome, it looks like the main differences are due to fever at Day 7 (and to a lesser extent, oxygen need at Day 7). I am not a clinical expert; however, it seems somewhat implausible to me that a strategy aimed at withdrawing antibiotics would help reduce fever at Day 7. In fact, my understanding was that the risk lied in the opposite direction. I therefore wonder whether this finding (a significant reduction in the proportion of patients experiencing strategy failure) might be due to chance and/or subject to some degree of bias. This needs to be explained/discussed in more details in the discussion.

**Answer 10.** We appreciate this comment of the reviewer. The outcome ‘strategy failure’ was a pre-defined composite outcome, covering different aspects of negative clinical outcomes that may be related to the antibiotic treatment strategy in the ED. Besides a change in management we chose to also include fever and oxygen need at day 7 to reflect prolonged or complicated illness. The a priori hypothesis was that withdrawing antibiotics could increase strategy failure. However, in reality we found a reduction of strategy failure. This is indeed unlikely to result from the reduction in antibiotic prescriptions in the low-intermediate risk children. However, a plausible explanation is the observed shift in antibiotic prescription from the low-intermediate towards the high risk children. Therefore we concluded in the Discussion section that “the reduction in strategy failure suggests that antibiotic prescription more appropriately targeted children that benefited from antibiotics”, reflected (among other things) by less fever and oxygen need at day 7. We elaborated more on this issue in the Discussion section of the revised manuscript, page 22, line 456-469. Moreover, we provided more details and discussion on the use of composite outcomes in the Methods (page 9, line 230) and in the Discussion (page 22, line 473), as suggested by reviewer 3.

Discussion, page 22, line 445-450

*“The other primary endpoint of not increasing strategy failure was met. Moreover, we observed a reduction in strategy failure, suggesting that antibiotic prescription more appropriately targeted children that benefited from antibiotics. This additional hypothesis was supported by our exploratory subgroup analysis, showing a safe reduction in antibiotic treatments in the low-risk group and a (non-significant) increase of prescriptions and a reduction of failures in the high-risk children. This suggests a shift in antibiotic prescriptions from the low-intermediate towards the high-risk children who had more clinical benefit more from the antibiotics.”*

Methods, page 9, line 229-234:

*“Since there is no single and objective measure of failure of antibiotic treatment strategy, we used a predefined composite outcome for strategy failure. This outcome was chosen to cover different aspects of strategy failure that are important in clinical practice and may be related to the initial treatment strategy at the ED.[20] It includes changes in treatment strategy of the child (secondary or switched antibiotic treatment and secondary hospitalization) as well as signs of prolonged or complicated disease (oxygen dependency or fever up to day 7 and complications).”*

Discussion, page 22, line 457-463:

*“We used a composite outcome to define strategy failure. Composite outcomes can be problematic, if the effect of the intervention is mainly driven by less important components.[20] In our study we found that a reduction in secondary antibiotic prescriptions was the main component in the high-risk children and in those in whom we could not calculate the risk score (Supplementary file S8). In low-risk children secondary prescription slightly increased, but without increasing oxygen need or fever at day 7, as proxies for disease severity. There was no increase in complications during the intervention phase. These observations show that our intervention was safe with reduced strategy failure on clinically important outcomes.”*

**Comment 11.** Please explain why "the data of patients included during the roll-out period" are thought to "carry most weight in the analyses" (cf lines 363-364).

**Answer 11.** In a stepped-wedge design estimates of the intervention effect are based on both the within-cluster and between-cluster information.[2] Before the roll-out period none of the hospitals used the intervention, and after the roll-out period all hospitals used the intervention. All children included before the roll-out were classified into one time-period (period 1), as well as those included after the time roll-out (period 9). Since we adjust for time-period, we can only estimate the between-cluster difference during the roll-out period. We agree this terminology was confusing and not necessary, so we have removed the sentence from the manuscript.

-Laurent Billot

**Reviewer #2**: van de Maat and colleagues have submitted a manuscript utilizing a stepped-wedge cluster randomized trial to assess the impact of a clinical decision rule on suspected lower respiratory tract infection in children less than five years of age. Utilizing 999 children enrolled in this study, the decision rule did not impact antibiotic prescribing but did result in less strategy failure. An exploratory analysis among low and intermediate risk children did identify a reduction in antibiotic use and no difference in strategy failure.

**1. Main concern:**

The conclusion was that the clinical decision rule did not reduce overall antibiotic prescription and less strategy failures occurred during the intervention. This is an appropriate conclusion based on the data. However, the authors intervention and power calculation were based on antibiotics not being prescribed in low and intermediate risk children but high risk children were enrolled and included in the analysis. The trial ended up assessing the use of the clinical decision rule in all patients and since likely a significant portion were high risk the impact on antibiotic use was not observed.

**1. Response to main concern:** We understand the concern of the reviewer and will explain the considerations behind our approach. We agree that our manuscript was not completely clear at this point. Our main aim was to reduce antibiotic prescriptions in children suspected of lower RTIs in the ED. This target population includes children with all different risk profiles, since at presentation in the ED their risk is unknown. In this complete group we introduced the decision rule as an intervention, with a differential effect on the risk groups: advice for no antibiotics in the low-intermediate risk children, advice for usual care in high-risk children. This differential treatment effect was also included in our sample size calculation: we estimated no effect on antibiotic prescriptions in the high-risk children, and a 10-15%-point reduction in the low-intermediate risk children. So our power calculation was also based on the complete target population of children suspected of lower RTIs, assuming a distribution of risk groups based on previous research. The calculated sample size would have been sufficient to prove a reduction of 10%-point in antibiotic prescription in the whole population, if 20% of children were high-risk, and if there was no effect on antibiotic prescription in this high-risk group. However, our exploratory subgroup analyses showed that in practice the high-risk population was larger (40%) and that there was a (non-significant) increase in prescriptions in the high-risk children. Even though this was not a significant increase, it may partly explain why we did not observe the reduction in the overall population. At the same time we observed the significant reduction in strategy failure especially in these high-risk children, suggesting that they benefited from the (increased) antibiotic prescriptions. However, our study was intended for non-inferiority of strategy failure and not powered for this subgroup analysis, and it could only be based on complete case analysis. Therefore we kept the conclusion to our primary analyses, and used the exploratory analyses to generate the hypothesis for the observed findings. Our study shows that an intervention can have unanticipated effects that are still relevant in the evaluation of the intervention. If it would have only included the low-intermediate risk children, we would have missed the effect the intervention had in the high-risk children.

We hope this addresses the concern of the reviewer, and we have elaborated on this issue in more detail in the Methods and the Discussion sections.

**Methods**, page 8, line 170:

*This target population included children with all different risk profiles, since at presentation in the ED their risk profile is unknown.*

And page 9, line 205

*The intervention was a decision rule-based treatment strategy (so-called decision rule) for all children suspected of lower RTIs in the ED, with a differential effect on risk groups.*

And page 11, line 248-254:

*We based our sample size calculation on the complete target population of children suspected of lower RTIs in the ED, including all risk groups. (…) The decision rule was expected to affect risk groups differently: we estimated no difference in antibiotic prescription in the high-risk patients, and a reduction of 10-15%-point in the low-risk and intermediate-risk patients, leading to an overall reduction of antibiotic prescriptions of 10%-point.*

**Discussion**, page 22, line 443-456:

*“We hypothesized that introducing a decision rule as an intervention would safely reduce antibiotic prescriptions in children suspected of lower RTIs in the ED. This target population included children with all different risk profiles, since at presentation in the ED their risk is unknown. The primary endpoint of reducing overall antibiotic prescription was not achieved. The other primary endpoint of not increasing strategy failure was met. Moreover, we observed a reduction in strategy failure, suggesting that antibiotic prescription more appropriately targeted children that benefited from antibiotics. This additional hypothesis was supported by our exploratory subgroup analysis, showing a safe reduction in antibiotic treatments in the low-risk group and a (non-significant) increase of prescriptions and a reduction of failures in the high-risk children. This suggests a shift in antibiotic prescriptions from the low-intermediate towards the high-risk children who had more clinical benefit from the antibiotics. Our power calculation was based on the complete target population of children suspected of lower RTIs, assuming a distribution of risk groups based on previous research. However, we observed a smaller proportion of low-risk children in our study population. The shift in antibiotic prescriptions towards high-risk children and the observed smaller proportion of low-risk children in our study may explain why we did not detect an overall reduction in antibiotic prescription. However, it must be noted that this was based on complete case analysis only and that our study was not powered for subgroup analyses.”*

Below are additional thoughts and criticism by section:

**Comment 2.** Title: The abstract and manuscript discuss decreasing antibiotics for lower respiratory tract infection and the title states pneumonia. While these are similar, I recommend a change from pneumonia to lower respiratory tract infection.

**Answer 2.** We thank the reviewer for this suggestion, and we have adjusted the title.

**Abstract**: Appropriate

**Introduction:**

**Comment 3.** The objective of the study was to lower antibiotic use in low or intermediate risk children, however all risk categories were enrolled so the study tested the impact of the clinical decision rule on children presenting with potential LRTI. To address just low or intermediate risk, only these patients should have been enrolled.

**Answer 3**. This comment is related to the main concern of the reviewer, so we refer to our answer to the main concern (comment 1).

**Methods:**

**Comment 4.** What number was considered a fever? This was stated in the supplemental protocol. For the readers it would be helpful to have this listed.

**Answer 4:** Fever was considered at a >38.5° Celsius. We have added this to the methods, page 8, line 168:

*“We included children aged one month to five years, that presented with fever (reported by parents or measured >38.5° Celsius at the ED) (…)”*

**Comment 5.** Page 7 line 136- What kind of otitis? Please be more specific as I'm assuming this is otitis media.

**Answer 5:** That is correct, we have added this.

**Comment 6.** After the intervention was implemented did all patients have the clinical decision rule utilized or only in patients that an informed consent was obtained? When was the informed consent obtained, before or after the use of the calculator?

**Answer 6:** The decision rule was utilized in all patients in whom informed consent was obtained. This was obtained before the use of the calculator. We added this to the methods, page 6, line 157:

*“Consent was obtained before calculating the predicted risk of the child.”*

**Comment 7.** Who performed the informed consent? Clinical coordinator, physician?

**Answer 7:** Informed consent was asked by the treating physician. We added this to the methods, page 6, line 154:

*“Written informed consent was obtained from the parents of all participants by the treating physician (…)”*

**Comment 8.** What constitutes a patient being ill? Was this based on just a clinicians generally belief or were there specific factors that put a patient in the ill category?

**Answer 8:** Ill appearance was based on the judgment of the treating clinicians. Although not being defined by specific criteria, in the development and validation of the Feverkidstool this characteristic appeared to be valid and consistent among different populations. We added this to the methods, page 10, line 212:

*“Ill appearance was based on the judgment of the treating clinicians. Although not being defined by specific criteria, in the development and validation of the Feverkidstool this characteristic appeared to be valid and consistent among different populations”*

Van den Bruel et al. published a systematic review on the diagnostic value of clinical predictors of serious infections in children, in which they evaluated different definitions of ‘ill appearance’, and they found similar results [3].

**Outcomes**

**Comment 9.** Were all antibiotics prescribed considered appropriate? If an antibiotic not recommended for pneumonia was prescribed were these patients excluded?

**Answer 9:** First, the reviewer address a major problem in treating pneumonia, i.e. that due to lack of a proper definition of bacterial pneumonia, the appropriateness of antibiotic prescriptions in pneumonia is hard to assess.[4] The absence of a gold standard for bacterial pneumonia impedes deciding upon appropriate treatment. This underlines the need to improve the clinical detection rate of those children who may benefit most from antibiotic treatment of bacterial pneumonia. To overcome the problem of the lacking gold standard, the focus of this study was to evaluate the impact of the decision rule on overall antibiotic prescription, not the appropriateness of the individual prescriptions.

This also answers the second question: we included all children fitting the inclusion and exclusion criteria, regardless of the type of antibiotics prescribed. The exclusion criterion ‘other identifiable infectious focus’ ensured that the antibiotics in the included children were prescribed to treat a respiratory tract infection.

We added a comment in the Introduction, page 6, line 118:

*The absence of a gold standard for bacterial pneumonia impedes deciding upon appropriate treatment. Therefore, we need to improve the clinical detection rate of those children who may benefit most from antibiotic treatment of bacterial pneumonia.*

**Comment 10.** Strategy failure was considered if an antibiotic was switched. If a patient had their antibiotic switched due to allergy or other adverse drug reaction was this considered a failure? Was the reason for switch documented?

**Answer 10.** The information on switching antibiotics was retrieved from telephone follow-up of parents. The intervention did not provide treatment advice during follow-up, and the reasons for switching antibiotics were not systematically recorded. So indeed, also if the antibiotics were switched for an allergy or adverse drug reaction, it was considered a failure.

We added this to the Methods, page 10, line 234-238 (also including the answer to the next comment #11):

*Changes in treatment strategy during follow-up were made without specific recommendations in the study protocol. Reasons for switching antibiotic prescription were not systematically recorded. We used a short follow-up period of one week, assuming that a secondary hospitalization within this timeframe was related to the respiratory illness. All secondary prescriptions and secondary hospitalizations were considered a strategy failure.”*

**Comment 11.** Strategy failure was also considered if a secondary hospitalization occurred. I assume this hospitalization had to be due to the same respiratory problem. The authors should specify this as some children could be hospitalized due to an unrelated problem.

**Answer 11.** We thank the reviewer for this observation. We used a short follow-up period of 1 week, therefore we assumed that a secondary hospitalization within this timeframe was related to the respiratory illness. All secondary hospitalizations were considered a strategy failure. We added this to the Methods, page10, line 238 (pasted in the answer to the previous comment #10). From 21 out of the 29 secondary hospitalizations we knew the indication: 11 were hospitalized for oxygen therapy and 8 for monitoring of the child, so our assumption seems valid.

**Comment 12.** Complications included parapneumonic effusion or empyema. Was there a certain size of an effusion that had to be present on a chest Xray? Were any effusion on a chest Xray considered a parapneumonic effusion? This should be better specified though with so few complications this point is very minor.

**Answer 12.** We did not specify the size of the parapneumonic effusions, and have added this information to the methods, page 10, line 242:

*“Complications were defined as the presence of pleura-empyema, parapneumonic effusion (any size), pulmonary abscess or respiratory insufficiency (need for mechanical ventilation) by day 7.”*

**Power calculation**

**Comment 13.** The power calculation was based on low and intermediate risk patients presenting with pneumonia though the study enrolled all patients.

**Answer 13**. This comment relates to the main comment (comment 1) of the reviewer and we refer to our reply at that point. In short, the power calculation was based on the whole population, but we assumed a differential effect of the intervention in the risk groups. In our power calculation the high-risk children were included, even though we assumed no effect on antibiotic prescriptions in this group. We have added this information to the methods section, page 10, line 248-254.

*“We based our sample size calculation on the complete target population of children suspected of lower RTIs in the ED, including all risk groups. (…) The decision rule was expected to affect risk groups differently: we estimated no difference in antibiotic prescription in the high-risk patients, and a reduction of 10-15%-point in the low-risk and intermediate-risk patients, leading to an overall reduction of antibiotic prescriptions of 10%-point.”*

**Comment 14.** The interim analysis changed the needed patients based on more patients in the low risk category receiving antibiotics with the assumption that a greater decrease in antibiotics would be observed. How were the authors able to determine the number of patients in the low risk group if not all patients had the necessary items (eg. crp) to calculate the risk?

**Answer 14.** It is true that there were missing data in the interim analyses. Interim re-estimates were based on the available data at that time. This was done before introduction of the intervention, so it was blinded to the outcome of the trial.

**Comment 15.** The authors mention that this analysis could evaluate non-inferiority in terms of strategy failure. What was the non-inferiority margin that was going to be used to determine if non-inferiority was present? Furthermore, this non-inferiority analysis was not performed so is this even needed to be stated?

**Answer 15.** We used a non-inferiority margin of 5%. We admit that our primary hypothesis was to evaluate non-inferiority in terms of strategy failure, and not superiority. We toned down the conclusions on strategy failure in the revised manuscript, describing non-inferiority instead of superiority.

Conclusion, page 25, line 506-509:

*“A clinical decision rule for childhood pneumonia did not reduce overall antibiotic prescription, but was non-inferior in terms of strategy failure. Exploratory analyses showed that the intervention reduced antibiotic prescriptions in low-intermediate risk children, and that it reduced overall strategy failures, suggesting improved targeting of antibiotics by the decision rule.”*

**Results:**

**Comment 16.** What is the break down in the intervention period of the number of patients in the different risk categories? This could be included in the table and in the pre intervention period just report on those that a score could be calculated.

**Answer 16.** We refer to Table 3 for the breakdown of the number of children in the low-intermediate versus high risk groups. We have added the breakdown of the number of low and intermediate risk children as a footnote to this table, page 21, line 408:

*“* 331/597 (55%) cases were complete of the pre-intervention population, of whom 172/331 (52%) in low-intermediate risk group (n=91 low risk; n=81 intermediate risk); 374/402 (93%) cases were complete of the intervention population, of whom 234/374 (63%) in the low-intermediate risk group (n=115 low risk; n=119 intermediate risk).”*

**Comment 17.** What is included in "demanding logistics?" Does this include being discharged before the patient could be enrolled since these patients were not as sick as those in the trial.

**Answer 17.** With demanding logistics we meant that it was too busy in the ED to enrol patients in the study. Doctors in the ED often are under time pressure, leaving insufficient time or attention to recruit patients for a trial, as has been acknowledged by previous paediatric ED implementation trials in ED trials, and mentioned in the Discussion (page 22, line 432). This indeed includes children being discharged before they could be enrolled.

We have rephrased the sentence in the Results section to clarify this (page 15, line 335).

*“The main reason for non-inclusion of patients was that the ED was too busy to enrol patients in the trial.”*

And we have rephrased the Discussion, page 22, line 432:

*“Next, not all eligible children could be included in the trial. Doctors in the ED often are under time pressure, leaving insufficient time or attention to recruit patients for a trial, as has been acknowledged by other paediatric ED trials.[5, 6]”*

**Comment 18.** AS stated before "ill appearance" should be more clearly described since so many children are reported to be ill appearing. I would include this as a footnote in table 1.

**Answer 18.** We have added this as a footnote in Table 1:

*“* Based on physician’s judgment (yes/no)”*

**Comment 19.** In table 1, the pre intervention type of antibiotics added up to 179. Did a patient receive 2?

**Answer 19.** We thank the reviewer for this observation, there was a minor error in these numbers, which we have corrected in the revised version. No children received two antibiotics, so all number add up correctly now.

**Comment 20.** Table 2- in the complications both pre and post intervention have it stated as 0%. In this situation since there is 1 patient in each group a 0.x% would be appropriate.

**Answer 20.** We agree and have adjusted this.

**Comment 21.** What percentage of the patients that would be considered high risk received antibiotics?

**Answer 21.** We refer to Table 3 for these numbers. In total 299 (159+140) children were considered high risk (pre-intervention + intervention of whom we could calculate risk group), of whom 75+83=158 received antibiotics, which is 53%.

**Discussion:**

**Comment 22.** The conclusion based on the data is fair but the study ended up including what were likely much more high risk patients in the study since all patient types were included which was not the objective of the study. I do realize a challenge was the lack of knowing the risk group in the pre-intervention phase.

**Answer 22.** This relates to the main concern (comment 1) of the reviewer, and we refer to our answer there.

**Comment 23.** The authors state the lack of CRP did not influence the primary analysis however not being able to compare the risk categories and only do this as an exploratory analysis was not the objective and intent of the study and the intervention. This leads to a conclusion about the decision rule that potentially underestimates the interventions impact.

**Answer 23.** This partly relates to the main concern. It should be noted that for the primary analyses the risk category was not necessary, as in the main models only age, gender, season, duration of fever and ill appearance were needed. Those variables had very few (<3%) missing data, so the lack of CRP did not influence our main results. However, it indeed did reduce the population size that could be analysed in our exploratory subgroup analysis. Therefore, we agree that our study may underestimate the effect of the intervention in the low-intermediate risk children. Future research should focus on populations with more low-intermediate risk children and with a higher prescription rate, where the impact of the intervention may be larger.

**Comment 24.** The authors state that the primary aim was to evaluate the overall impact of a decision rule on antibiotic prescription and strategy failure. The authors stated objective was to "safely reduce antibiotic prescription in children under five suspected of a lower RTI at the ED, by withholding antibiotics in children at low or intermediate risk of bacterial pneumonia, as predicted by the Feverkidstool." The authors should remove first sentence of the section "Interpretation of results…" and focus on what the main objective was. The results were more directed toward the overall impact and not those with just low or intermediate risk.

**Answer 24.** We thank the reviewer for this suggestion and have adjusted the first sentence of the ‘Interpretation’ section of the Discussion, page 22, line 442:

*“Our primary aim was to safely reduce antibiotic prescription in children under five suspected of a lower RTI at the ED.”*

**Comment 25**. The authors discuss low and intermediate risk patients but present limited data on the number of patients in each category.

**Answer 25.** This relates to comment 21 of this reviewer, and we believe the number of patients in each risk group is now more clear in the footnote of Table 3.

Overall, this manuscript is **interesting**. The primary objective is not truly evaluated based on the patients enrolled and the results presented. Because of these results, the study did not substantially assess the hypothesis of using the decision rule to decrease antibiotic use, though **it appears the decision rule has benefits in both reduction and strategy failures.**

**Reviewer #3:** This is an **important** study using a stepped-wedge cluster RCT design to evaluate an intervention to safely reduce antibiotic prescriptions. During the preintervention phase usual care is provided to childhood pneumonia patients in 8 hospitals in The Netherlands. This is followed by an intervention phase during which a validated clinical prediction model (clinical characteristics and C-reactive protein) is used to guide antibiotic prescription in children with uncomplicated pneumonia. The co-primary outcomes in the trial are antibiotic prescription rate and strategy failure comprising secondary antibiotic prescription or hospitalisation, persistent fever or oxygen dependency and complications.

**Comment 1.** The abstract states that the trial could not be blinded and the same is repeated within the text in the method section without further elaboration as to why blinding could not be achieved. This statement on failure to implement blinding can be strengthened in the methods section of the manuscript by explaining why it was not possible to achieve blinding.

**Answer 1.** We thank the reviewer for this suggestion. Since the nature of the intervention was the implementation and active use of a decision rule in clinical care, including treatment advice, this could not be blinded. We have added further explanation on blinding in the Methods section, page 8, line 189.

*“The trial could not be blinded, because the intervention was the implementation and use of a decision rule by clinicians in the ED, including a treatment advice based on the risk score that had to be calculated per child.”*

**Comment 2.** The abstract reports that 8 clusters (hospitals) were allocated to sequences of treatment but does not report the number of hospitals randomised to each sequence of treatment and the number of sequences.

Similarly, this information is not contained in the body of the manuscript.

**Answer 2.** We agree that this could be made more explicit. We have now rephrased the sentence in the Abstract to:

*“Hospitals were randomly allocated to one sequence of treatment each, using computer randomization.”*

And in the Methods section we explained this in more detail, page 6, line 145:

*“A cluster consisted of one hospital, that was randomized to one sequence of treatment. One hospital was randomised to one sequence of treatment, resulting in 8 sequences.”*

We also refer to the diagram in Figure 1 (was supplementary file S1 in first version of the manuscript), which graphically shows all the hospitals and sequences of treatment. We have included this graph in the main body of the manuscript.

**Comment 3.** The introduction is well written with sufficient detail to justify the conduct of the study. It also contains the objectives for the trial but does not give a rationale for a stepped wedge design.

**Answer 3.** That is true. Because this is a relatively new study design, providing the rationale for choosing this design is rather wordy. Therefore we chose to describe this in the Methods section of our manuscript (page 6, line 139-142) instead of in the introduction:

*“The stepped-wedge design was preferred as in general smaller sample sizes are needed than in conventional cluster randomized trials. Because clusters act as their own controls, the intervention effect can be estimated from both between and within cluster comparisons. The sequential implementation of the intervention was superior to a conventional before-after design, given the incorporation of time effects.”*

**Comment 4.** The authors do not describe their attempts (if any) at allocation concealment. It is noteworthy that allocation concealment can be implemented even in situations where blinding is not feasible and that individual recruitment into cluster trials without concealment of allocation (or blinding) increases the risk of selection bias.

**Answer 4.** Since in our trial allocation to a treatment sequence was based on clusters, allocation concealment meant that the clusters were unaware of their treatment sequence until after recruitment (item 9 of the Consort checklist of SW-CRT). This was achieved, since allocation of sequences was performed in July 2017, after recruitment of all hospitals. This was mentioned only in the Methods section (page 8, line 184), but we also made this more explicit in the Results section, page 15, line 328:

*“All hospitals that were assessed for eligibility were recruited (n=8), after recruitment all eight were randomized to a treatment sequence and included in the analyses (Figure 1).”*

Allocation concealment at individual level was not possible, because of the randomization at cluster level and the impossibility of blinding for the intervention. In theory, if research staff were available on all study sites 24/7, patients could be recruited for the study before the medical consultation that includes the risk assessment. However, this is not feasible in practice. We know that only a minority of patients declined participation after they were asked for consent (10% of non-included patients with known reasons, Figure 2) and that this proportion was similar in the pre-intervention and intervention phase. Therefore, we believe that the fact that allocation was known, did not result in selection bias at individual level. We added this comment to the Discussion, page 21, line 434-437:

*“Comparison of the included and non-included children showed that we included severely ill children more frequently. This was the same in both phases of the study, and the rate of eligible children that declined participation was also stable over the study phases. Therefore, we believe there was no selection bias introduced by a lack of allocation concealment at individual level.”*

**Comment 5**. More details are required about the randomisation, recruitment and assignment of clusters to allow the reader ascertain the risk of bias. The authors should provide more details about the statistician who generated the random sequence - independence of the statistician, knowledge of cluster identities, and any other role in the trial. Who recruited the clusters? Who assigned the clusters to the sequence?

**Answer 5.** This relates to the previous comments, and we believe this is more clear now we have moved supplementary file S1 to the main body of the manuscript as Figure 1. Regarding the statistician and the person recruiting the hospitals, we added more details to the Methods section, page 8, line 180 and 185-187:

*“The hospitals were recruited by the principal investigator (RO).”*

*“The statistician was involved as an advisor in the trial and was based in the Erasmus MC. He knew the names of the other participating centres at randomization, but had no further knowledge of these hospitals or relation to the local researchers.”*

**Comment 6.** The trial has two co-primary outcomes: antibiotic prescription and strategy failure (that comprised five components - two of the five components i.e. secondary antibiotic prescription and hospitalisation are based on clinician judgment). The authors refer to antibiotic prescription outcome as a rate in the abstract, while in actual terms this is a proportion and not a rate. The authors overcome some of the difficulties associated with defining and reporting composite outcomes by providing a consistent definition of strategy failure in the abstract, methods and results. The authors also present data for all composite components useful for determining whether a similar effect occurred for all components of the composite. (Cordoba et al. BMJ 2010; 341:c3920 doi:10.1136/bmj.c3920) The following changes could further enhance the reporting of the composite outcome. Providing a rationale for the composite outcome including clinical importance of the components will help with

interpretation of the results. At present the discussion and interpretation of the significant findings of reduction in strategy failure seem to ignore the fact that this was a composite outcome and that this outcome was mainly driven by secondary antibiotic prescription. Please refer to Cordoba 2010 on interpreting and discussing effects based on composite indicators.

**Answer 6.** We thank the reviewer for these suggestions to improve the reporting of the composite outcome strategy failure. First, we have elaborated more on the clinical importance in the Methods section, page 9, line 229-234:

*“Since there is no single and objective measure of failure of antibiotic treatment strategy, we used a predefined composite outcome for strategy failure. This outcome was chosen to cover different aspects of strategy failure that are important in clinical practice and may be related to the initial treatment strategy at the ED.[ref] It includes changes in treatment strategy of the child (secondary or switched antibiotic treatment and secondary hospitalization) as well as signs of prolonged or complicated disease (oxygen dependency or fever up to day 7 and complications).”*

Moreover, we discussed the different components of the composite outcome in the Discussion section, page 21, line 457-463, referencing to the study by Cordoba et al:

*“We used a composite outcome to define strategy failure. Composite outcomes can be problematic, if the effect of the intervention is mainly driven by less important components.[ref] In our study we found that a reduction in secondary antibiotic prescriptions was the main component in the high-risk children and in those in whom we could not calculate the risk score (Supplementary file S8). In low-intermediate risk children secondary prescription slightly increased, but without increasing oxygen need or fever at day 7, as proxies for disease severity. There was no increase in complications during the intervention phase. These observations show that our intervention was safe with reduced strategy failure on clinically important outcomes.”*

**Comment 7.** The authors are **transparent** in reporting the change in sample size calculations after the initial protocol. They also adopt a well described approach for calculating sample size in stepped wedge trials. The sample size calculation as reported is difficult to replicate because details on the number of clusters and clusters allocated at each sequence, cluster size, and ICC or coefficient of variation or assumptions made about these parameters are not presented in the sample size justification. Was an allowance for variation in cluster size made because from supplementary table 6 there was considerable variation in the final cluster sizes?

**Answer 7.** We have provided more details about these parameters in the revised manuscript. As already mentioned before, we assumed 1 cluster per sequence. We assumed different cluster sizes (small, intermediate and large clusters) and three-level seasonal variation in inclusion of children. We have listed the exact assumptions in Supplementary file 3 and have added this information to the Methods, page 10, line 256:

*“We assumed different cluster sizes (small, intermediate and large clusters) and three-level seasonal variation in inclusion of patients. All assumptions are listed in Supplementary file 3.”*

**Comment 8.** The sample size justification contains the terms, interim analysis, risk reduction, superiority and non-inferiority. The aim of the study as stated in the introduction section was to safely reduce antibiotic prescription. The sample size calculation should be based on this aim and not to show superiority or non-inferiority as implied in the final sentences in the sample size justification. The authors should consider revising the use of the terms superiority and non-inferiority in their sample size justification, because retaining these terms in the sample size justification will require adopting a different approach to estimating sample size. In the absence of an intracluster correlation coefficient, I found the assumption that a power of 90% for independent data would equate to 80% power for correlated data to be a strong assumption that needs justification.

**Answer 8.** We refer also to our response to comment 15 of reviewer #2:

We used a non-inferiority margin of 5%. We admit that our primary hypothesis was to evaluate non-inferiority in terms of strategy failure, and not superiority. We toned down the conclusions on strategy failure in the revised manuscript, describing non-inferiority instead of superiority, page 24, line 506:

*“A clinical decision rule for childhood pneumonia did not reduce overall antibiotic prescription, but was non-inferior in terms of strategy failure. Exploratory analyses showed that the intervention reduced antibiotic prescriptions in low-intermediate risk children, and that it reduced overall strategy failures, suggesting improved targeting of antibiotics by the decision rule.”*

**Comment 9.** The primary analysis in this paper is based on a multilevel logistic regression model clustered by eight hospitals and adjusting for time as a fixed effect. The correlation of observations in the clusters is accounted for by inclusion of the hospitals as random effects in the multilevel model. This is one of the recommended approaches for analysing intervention effect in stepped wedge studies. The analysis of stepped wedge trials using mixed effect models requires strong assumptions about correlation of observation within each cluster. These assumptions may be inappropriate when the number of clusters is small as is the case in the current study. (Thompson et al. Stats Med. 2018; 37(16) 2487-2500) The authors should explain the potential impact of the few clusters in the trial on the results obtained from analysis approach that is suited for analysis involving a large number of clusters.

**Answer 9.** Due to the low number of clusters included in the analysis there may be an underestimation in the standard error of the treatment effect. Unfortunately we could not apply the suggested non-parametric test proposed by the reviewer as sample sizes per time period were very small and we would be unable to reliably estimate the intervention effect per time period with associated standard error as required by the proposed test (see supplementary file S7 for antibiotic prescription per cluster and time period, provided in this document at page 5.)

**Comment 10.** The sensitivity analysis section does not provide details on the type of imputation that was done for the first of the four sensitivity analyses apart from the statement that covariates with >10% missing data were imputed. The second sensitivity analysis appear to be based on single imputation. The authors should consider commenting on the limitations of their approaches to imputing missing data.

**Answer 10.** We agree that this issue was not totally clear in the Methods section. We used the multiple imputed database for all primary and sensitivity analyses, where we imputed all predictor variables with >10% missing data and without imputing the outcome variables antibiotic prescription and strategy failure. However, for the sensitivity analysis assessing the effect of missing data for strategy failure, we indeed used single imputation for that variable, assuming failure in all children with missing data in 1 dataset, and assuming no failure in all children with missing data in another dataset. All other variables in the dataset were from the same multiply imputed dataset that was used in the primary and other sensitivity analyses. We have added this information to the Methods section, page 13, line 314-319:

*“The imputation model included all of the variables needed for the primary and sensitivity analyses, as well as additional information on diagnosis, treatment, disposition and follow-up. Outcome variables (antibiotic prescription and strategy failure) were not imputed, except for the sensitivity analysis evaluating the effect of loss-to-follow-up on the outcome strategy failure. In this sensitivity analysis we used single imputation only for the outcome variable strategy failure, assuming failure if the variable was missing in one dataset versus no failure in another dataset.”*

**Comment 11.** The multiple imputation assumed data were missing at random. A sensitivity analysis of departure from missing at random assumption will be helpful in assessing the robustness of the reported results following multiple imputation.

**Answer 11.** We indeed assumed data to be missing at random and pre-planned a sensitivity analysis in case covariates had >10% missing data. In practice, three out of five needed covariates were complete (age, sex and season), of fever duration only 7/999 (<1%) children had missing data and of ill appearance we had 27/999 (3%) missing data. Therefore we did not perform the pre-planned sensitivity analysis in the manuscript (page 18, line 367). For the reviewer’s information, we now performed a sensitivity analysis of the primary outcomes in the intention-to-treat population, departing from the assumption that ill appearance was missing at random, since this variable had most missing data. As this table shows, this leads to very minor changes, supporting the robustness of the results. We did not include these data in the revised manuscript.

| **Sensitivity analysis** | Adjusted OR (95% CI) | Adjusted OR (95% CI) | Adjusted OR (95% CI) |
| --- | --- | --- | --- |
|  | *Missing = imputed* | *Assumption missing = ill* | *Assumption missing = not ill* |
| - Antibiotic prescription | 1,07 (0,57-2,01) | 1,06 (0,56-1,98) | 1,08 (0,58-2,04) |
| - Strategy failure | 0,53 (0,32-0,88) | 0,54 (0,32-0,89) | 0,53 (0,32-0,87) |

**Comment 12.** The results are **appropriately presented** based on the analysis approach that was adopted and the recommendation for reporting contained in the CONSORT extension for stepped wedge cluster RCT. Results are presented for both intention-to-treat and per protocol analysis.

**Answer 12.** Thank you.

**Comment 13.** The authors state in the discussion "Clinical characteristics of children with missing Feverkidstool variables were comparable to those with complete information. This supports the assumption that missing data were at random, with no major bias to our subgroup analyses". Based on literature on missing data, this is an untestable assumption because the data we would need to test it is missing (Carpenter & Kenward. 2013. Multiple imputation and its applications. John Wiley & Sons, UK)

**Answer 13.** It is true that the assumption that missing Feverkidstool variables were missing at random cannot be tested, so we removed this sentence from the Discussion and rephrased it to: (page 21, line 432)

*“This may have introduced some bias in the subgroup analyses.”*

**Comment 14.** A more detailed discussion of the authors statement that they "included severely ill children more frequently" is warranted because this could point to selection bias.

**Answer 14**. This is partly related to comment 4 of the reviewer. The pattern of including the more severely ill children was the same in the pre-intervention and in the intervention phase. Therefore, we believe this did not introduce selection bias to our results. We have added this comment to the Discussion, page 21, line 435.

*“Comparison of the included and non-included children showed that we included severely ill children more frequently. This was the same in both phases of the study, and the rate of eligible children that declined participation was also stable over the study phases. Therefore, we believe there was no selection bias introduced by a lack of allocation concealment at individual level.”*

**References:**

1.	European Medicines Agency. ICH E9 statistical principles for clinical trials 1998 [15-11-2019]. Available from: <https://www.ema.europa.eu/en/documents/scientific-guideline/ich-e-9-statistical-principles-clinical-trials-step-5_en.pdf>.

2.	Hussey MA, Hughes JP. Design and analysis of stepped wedge cluster randomized trials. Contemp Clin Trials. 2007;28(2):182-91. Epub 2006/07/11. doi: S1551-7144(06)00063-2 [pii]

10.1016/j.cct.2006.05.007. PubMed PMID: 16829207.

3.	Van den Bruel A, Haj-Hassan T, Thompson M, Buntinx F, Mant D, European Research Network on Recognising Serious Infection. Diagnostic value of clinical features at presentation to identify serious infection in children in developed countries: a systematic review. Lancet. 2010;375(9717):834-45. PubMed PMID: 20132979.

4.	Bradley JS, Byington CL, Shah SS, Alverson B, Carter ER, Harrison C, et al. The management of community-acquired pneumonia in infants and children older than 3 months of age: clinical practice guidelines by the Pediatric Infectious Diseases Society and the Infectious Diseases Society of America. Clin Infect Dis. 2011;53(7):e25-76. Epub 2011/09/02. doi: cir531 [pii]

10.1093/cid/cir531. PubMed PMID: 21880587.

5.	Lacroix L, Manzano S, Vandertuin L, Hugon F, Galetto-Lacour A, Gervaix A. Impact of the lab-score on antibiotic prescription rate in children with fever without source: a randomized controlled trial. PLoS One. 2014;9(12):e115061. Epub 2014/12/17. doi: 10.1371/journal.pone.0115061

PONE-D-14-30849 [pii]. PubMed PMID: 25503770; PubMed Central PMCID: PMC4263728.

6.	van Houten CB, Cohen A, Engelhard D, Hays JP, Karlsson R, Moore E, et al. Antibiotic misuse in respiratory tract infections in children and adults-a prospective, multicentre study (TAILORED Treatment). Eur J Clin Microbiol Infect Dis. 2019;38(3):505-14. Epub 2019/02/02. doi: 10.1007/s10096-018-03454-2

10.1007/s10096-018-03454-2 [pii]. PubMed PMID: 30707378.
