# Supplementary material for: Evaluation of a clinical decision rule to guide antibiotic prescription in children with suspected lower respiratory tract infection in The Netherlands: A stepped-wedge cluster randomised trial
Source: PLoS Med. 2020 Jan 31;17(1):e1003034. doi: 10.1371/journal.pmed.1003034 (PMC6993966; doi:10.1371/journal.pmed.1003034)
Supplement: Attachment — Submitted filename: Response to reviewers_231219.docx [file pmed.1003034.s011.docx]

Manuscript reference number: PMEDICINE-D-19-03060R1

Title: A clinical decision rule guiding antibiotic prescription in children suspected of lower respiratory tract infections in The Netherlands: a stepped-wedge, cluster randomized trial

Dear Louise Gaynor-Brook,

We are very pleased to hear that you have decided to accept our manuscript for publication in PLOS Medicine. We think we could address the remaining comments of reviewers #2 and #3 as outlined in our response below. We have revised the manuscript accordingly. Because of the required additions to the abstract, the word count of the abstract in the revised manuscript is now exceeding 500 words. In the submission form we have shortened the sentence on complications, resulting in a word count of 500 words.

Kind regards, on behalf of all co-authors,

Josephine van de Maat

Erasmus MC – Sophia Children’s Hospital

Rotterdam, The Netherlands

Dr. R. Oostenbrink

Paediatrician – epidemiologist

------------------------------------------------------------

**Requests from Editors:**

**Comment 1.** Please put reference brackets before the full stop (or any punctuation), and after a space at the end of the word/sentence. **Answer 1:** Done.

**Comment 2.** Please revise your title according to PLOS Medicine's style. We suggest "Evaluation of a clinical decision rule to guide antibiotic prescription in children suspected of lower respiratory tract infections in The Netherlands: a stepped-wedge, cluster randomized trial".

**Answer 2.** We thank the editor for this suggestion and have adjusted the title accordingly.

**Comment 3.** Data Availability: Thank you for providing a link to the repository and a point of contact (not an author) for data access. Please explain the reasons for restricting data access to between 12 months and 10 years after publication. PLOS Medicine requires that the de-identified data underlying the specific results in a published article be made available at the time of article publication, provided it is legal and ethical to do so. Please confirm that data will be made available at the time of publication, and not at 12 months following publication.

**Answer 3.** Given the progress of other ongoing publications we can agree with the data being available at time of publication. The limit of 10 years following publication is the timeframe in which we can guarantee the storage of and access to the data in this repository.

**Comment 4.** Abstract Background: Please expand upon the context of why the study is important.

**Answer 4.** We have elaborated more on the importance of the study in the abstract, line 30-34.

*“Optimizing the use of antibiotics is a key component of antibiotic stewardship. Respiratory tract infections are the most important reason for antibiotic prescription in children, even though most of these infections in children under five years are viral. This study aims to safely reduce antibiotic prescriptions in children under five suspected of a lower respiratory tract infection at the emergency department (ED), by implementing a clinical decision rule.”*

**Comment 5.** Abstract Methods and Findings:

Please include more detail on the setting (e.g. EDs in cities in the Netherlands; types of hospital).

Line 54 - please clarify what constitutes ‘usual care’ e.g. according to what clinical guidelines

Please provide the number of children included in each group (based on the sequence of treatment available at the ED visited)

Please add a sentence to the abstract to mention the two complications quoted around line 390.

In the last sentence of the Abstract Methods and Findings section, please add “ potentially affecting the power of our study” with relation to the longer baseline period.

**Answer 5**. We have adjusted the Methods and Findings section of the abstract according to these comments.

**Comment 6**. Please begin your Abstract Conclusions with "In this study, we observed ..." or similar.

Line 76 - please revise both instances of ‘less’ to ‘fewer’

**Answer 6**. Done.

**Comment 7.** Author Summary:

Line 88 - please revise ‘part’ to ‘number’

Line 98 - please revise both instances of ‘less’ to ‘fewer’; please consider another term for ‘failed the initial treatment’

Line 102 - please replace ‘First of all, this means that…’ with ‘We observed that’

**Answer 7.** Done. We have rephrased the term ‘failed the initial treatment’ to (line 87):

*“It seemed that a higher number of children responded well to their initial treatment”*

**Comment 8.** Methods:

Please provide more justification in the Methods (as you have in your rebuttal letter) for the adjustment to sample size from 1100 to 900 children.

**Answer 8.** We elaborated more on the adjustment of the sample size in the Methods section, line 151-154:

*“Interim analysis after the first year of data collection (but before implementation of the intervention) showed substantially higher antibiotic prescription rates than anticipated. Based on the distribution of risks and actual antibiotic prescription rates during the pre-intervention period, the sample size was adjusted from 1100 to 900 children, which is also reported in the NTR.”*

**Comment 9.** Line 162 - Please cite the supplementary file containing your CONSORT checklist.

**Answer 9.** We have cited this file as supplementary file S3 and have adjusted the numbering of the subsequent supplementary files.

**Comment 10.** Line 169 - Please include more detail on the setting (e.g. EDs in cities in the Netherlands).

**Answer 10.** For the description of setting we refer to line 171 – 175, where we have added more details in the revised version of the manuscript:

*“We included eight hospitals in six cities of the south-west and central area of The Netherlands: (a) where paediatricians were responsible for the children presenting at the ED, (b) with varying ED sizes (range 500 – 13,000 yearly paediatric ED admissions), (c) from diverse geographic and demographic regions (inner-city versus mixed rural/urban) and (d) of different hospital types (tertiary versus general).”*

Some of these details are also presented in Fig 1.

**Comment 11.** Line 194 - Please clarify what constitutes ‘usual care’ e.g. according to clinical/local guidelines?

**Answer 11.** We have improved the explanation of usual care, line 190-192:

*“During the pre-intervention phase all children received usual care. Usual care consisted of triage by a nurse including the routine measurement of vital signs, followed by a clinical assessment and initiation of therapy by a physician, according to the Dutch and NICE guidelines for febrile children. [refs 17,18 in manuscript]”*

**Comment 12.**

Line 211 - please define APLS

Line 261 - please amend to Supplementary File S1

Line 337 - please revise ‘less’ to ‘fewer’

**Answer 12.** Done.

**Comment 13.** Tables.

Tables 2 & 3 - When a p value is given, please specify the statistical test used to determine it in the legend.

Table S5 - Please specify the statistical test used to determine statistical significance

**Answer 13.** In the Tables 2 and 3 the p-values are based on multivariable logistic regression. In supplement S5 comparisons of characteristics between included and non-included children are based on t-tests for continuous outcomes and chi-squared tests for categorical outcomes. We have added this information to the legends of the tables.

**Comment 14.** Discussion

Please remove all subheadings from within the Discussion i.e. ‘principal findings’ and so on

Please reorganize the Discussion as follows: a short, clear summary of the article's findings; what the study adds to existing research and where and why the results may differ from previous research; strengths and limitations of the study; implications and next steps for research, clinical practice, and/or public policy; one-paragraph conclusion.

**Answer 14.** Done.

**Comment 15**. Protocol

Supplementary Files 1 and 2 correspond to the final protocol and original protocol respectively. Please amend.

**Answer 15**. Done.

**Comment 16**. References: Please ensure all journal titles are appropriately formatted and capitalised e.g. BMJ [ref 20]

**Answer 16.** Done.

**Comment 17.** CONSORT statement - please format the final column of your checklist as some information appears to have been lost in the .pdf file provided. Please provide section and paragraph numbers.

**Answer 17.** This is a fixed fill-in pdf-document, so there is not much space in the column. We have adjusted the column, referring to the sections with the first letter of the section (e.g. A for abstract, I for introduction etc.) followed by the paragraph number.

**Comments from Reviewers:**

**Reviewer #2:** The authors have thoroughly addressed my reviewer comments. I applaud the authors for the detail and time spent in responding to my concerns. I still have 2 thoughts for consideration

**Comment 1.** The authors' state on page 5 line 118, "The absence of a gold standard for bacterial pneumonia impedes deciding upon appropriate treatment." I disagree with this statement. While the true gold standard for diagnosing pneumonia would be sampling the lung, this will never be done for many important reasons. However, the common pathogens to cause community-acquired pneumonia in the age-group studied (eg. Streptococcus pneumoniae) provide us the ability to make determinations of which antibiotics are inappropriate in the population included in this study. The guideline referenced in the response to the reviewers provide recommendations for antibiotics in the outpatient setting. For example, it would be inappropriate to prescribe a quinolone and/or azithromycin. Some experts would suggest prescribing cefdinir for pneumonia would also be inappropriate. Regardless, I think the authors' goal of reducing total antibiotic prescribing is important and they should just note that appropriateness of the antibiotic was not determined.

**Answer 1.** We agree with the reviewer that distinctions between appropriate and inappropriate empiric treatment can be made when it comes to types of antibiotics. In our statement in the introduction we were referring to the fact that the actual determination of pathogens in blood or sputum samples is often not feasible in children. Following the remarks of the reviewer we have rephrased the sentence in the revised introduction, line 109:

*“In the absence of a gold standard for bacterial pneumonia we need to improve the clinical detection rate of those children who may benefit most from antibiotic treatment of bacterial pneumonia.”*

**Comment 2.** Is the strategy failure within 7 days of starting or completing the antibiotic? I think it is from starting the antibiotic based on other statements in the manuscript but I think it would be helpful for the reader to have it listed in the outcome paragraph.

**Answer 2.** Strategy failure is within 7 days after the initial ED visit, in which antibiotics may be prescribed or not. If antibiotics were prescribed, failure would indeed be defined as within 7 days of starting the antibiotic. We have added this point to the methods section, line 222:

*“Primary outcomes were antibiotic prescription (yes/no) at ED discharge and strategy failure within 7 days after the initial ED visit (yes/no).”*

**Comment 3.** The authors should specifically state in the outcomes that changing of antibiotics due to adverse drug reaction was considered a treatment failure.

**Answer 3.** We added this statement to our revised manuscript, line 235:

*“Switching of antibiotics due to adverse drug reaction was considered a strategy failure.”*

**Comment 4.** The authors reported that they did non inferiority but I did not see the non-inferiority margin of 5% listed in the statistical analysis portion of the manuscript.

**Answer 4.** We have added this to the statistical analysis section of the revised manuscript, line 262-265:

*“This number was also sensitive to shown non-inferiority of the intervention in terms of strategy failure with a non-inferiority margin of 5%: it could detect a twofold increase of secondary hospitalization (the part of strategy failure with available baseline data, 5% at time of original sample size calculation) with a power of 0.8 and alpha 0.05.”*

**Reviewer #3:** The authors have responded adequately to most of the comments raised in the review. The following specific comments need to be addressed further.

**Comment 1.** What was the basis for assuming that 90% power under independence equates to 80% power in a scenario of non-independence? This question was not addressed in the authors' responses.

**Comment 2.** Will the figure that contains the range of ICCs used in the sample size calculation (provided in response to reviewer's comments) be included in the final manuscript? If not then it will be useful to write these ranges in the sample size section to allow readers to independently replicate the sample size calculation. I reckon that although the ICC is unknown these ranges were used as plausible estimates. If they are not provided then a reader cannot follow the sample size calculation.

**Answer to both comments.**

At the time of the original protocol the assumption of 90% power under independence equating to 80% power under non-independence seemed reasonable, in the absence of information on the ICC. We have tested this assumption in the post-hoc sensitivity analysis of the sample size calculation, showing little variation in sample size at varying ICC levels. This figure or ICC ranges will not be included in the methods of the final manuscript, because it was a post-hoc sensitivity analysis that was not described in the original protocol and analysis plan. In our previous revision this information was not included but we had suggested in the rebuttal to the editor to add a comment on this in the Discussion section. Following the current comments of the reviewer, we have now included the following comment in line 431-433, with addition of the ICC ranges:

*“Post-hoc sensitivity analysis of the sample size calculation showed that our study was sufficiently powered (power of 0.8), also when accounting for clustering at varying ICC (range 0.01 – 0.26) and adjusted for multiple testing.”*

**Production Issues:**

**Figures**

**Comment 1.** Please ensure that all main figures are in .tif or .eps format. For more information about how to convert your figure files, please see our guidelines. **Answer**: done.

**Comment 2**. The smaller text in Fig 1 is below our minimum font size of 8pt and will be difficult to read when published. Please remake the text in question at a larger size or take steps to make the text readable.

**Answer:** As far as I could see the minimum font size was 8pt; but I have adjusted all text in Fig 1 to size 10pt in the latest version.

**Comment 3.** Please remove embedded figures from your manuscript file and ensure the most up to date versions of your figures are uploaded to our submission system as separate TIF or EPS files. Please do not remove the figure legends and captions. **Answer:** done.

**Comment 4.** Please ensure that all main figures are referenced in the manuscript as Fig 1, Fig 2, etc. (including capitalization), rather than Figure 1, Figure 2, etc. Please note, however, that the file names themselves must not include the space, i.e. Fig1.tif, Fig2.tif. **Answer:** done.

**Comment 5.** Please ensure that all main figure legends are placed after the paragraph containing their first in-text citation. **Answer:** done.

**Metadata**

**Comment 1**. In accordance with PLOS' data policy, please ensure that your Data Availability Statement in the submission form clearly identifies how readers can access your data. Note that it will be typeset as it is written so please ensure it is in complete sentences and appears as you would like it to in the published version.

**Answer:** done.

**Article body**

**Comment 1.** Please make use of our title page guide and reformat accordingly.

**Answer**: done.

**Comment 2.** Please indicate the corresponding author in the byline by placing an asterisk (*) after their affiliation number. **Answer**: done.

**Comment 3.** Please include the corresponding author's email address on the title page of your manuscript, indicated by an asterisk (*). Only an asterisk and the email address itself are required. **Answer**: done.

**Comment 4.** Please remove author degree titles from the byline of your article file. **Answer**: done

**Comment 5.** Please remove the funding information from your manuscript file. The published version is pulled automatically from the “Financial Disclosure” field in the submission form and must not be duplicated. Please therefore take this opportunity to check that the information in this field is correct and completed in full. **Answer**: Done.

**Comment 6.** Please remove the Competing Interests section from your manuscript file. The published version is pulled automatically from the "Competing Interests" field in the submission form and must not be duplicated. Please therefore take this opportunity to check that the information in this field is correct and completed in full.

**Answer**: Done.

**Comment 7.** Please remove your Author Contributions section from your manuscript file as this information is automatically pulled from the authors section of our submission form and must not be duplicated. Please therefore take this opportunity to check that the information in this field is correct and completed in full.

**Answer:** Done.

**Comment 8.** Please also remove all metadata-related sections from the end of your manuscript (i.e. ethics, patient involvement, protocol, etc.). Please ensure your ethics statement is included in your methods section.

**Answer**: Done.

**References**

**Comment 1.** Please put all reference citations in square brackets separated by either commas or dashes with no spaces, e.g. [1,2,3] or [1-3].

**Answer**: Done.

**Comment 2**. Please ensure your references are in the style of PLOS Medicine (ICMJE/Vancouver style). The reference style guide can be found here [link in email]. You can also find a downloadable EndNote file in these guidelines to assist you. Please do a manual check as well, as the EndNote file does not catch everything. Please note that the references will be typeset as they appear so please ensure the format is consistent.

**Answer**: Done.

**Supporting Information**

**Comment 1.** Please ensure that all Supporting Information files use the following format verbatim throughout the manuscript (including legends and all in-text citations): S1 Fig, S1 Table, S1 Text, S1 Data, etc. Each file type should start at 1 and increase numerically. Please note, however, that the file names themselves must use an underscore rather than a space, i.e. S1_Fig.tif, S1_Table.xlsx, S1_Text.doc, S1_Data.xlsx, S1_PRISMA_Checklist.docx, etc. This means S5 Table should be S1 Table, etc.

**Answer:** done.

**Comment 2.** Please note that your submission cannot contain any references to generic "supporting information" files and instead must refer to specific files using our naming convention (S1 Fig, S1 Table, S1 Text, S1 Data, etc.)

**Answer:** done.

**Comment 3.** Please ensure that each supporting information file has a legend at the end of the manuscript file, after the Reference list.

**Answer:** done.
